# Size dependency of gold nanoparticles interacting with model membranes

**DOI:** 10.1038/s42004-020-00377-y

**Published:** 2020-09-17

**Authors:** Claudia Contini, James W. Hindley, Thomas J. Macdonald, Joseph D. Barritt, Oscar Ces, Nick Quirke

**Affiliations:** 1 Department of Chemistry, Molecular Sciences Research Hub, Imperial College London, White City Campus, Wood Lane, W12 0BZ London, UK; 2 Institute of Chemical Biology, Molecular Sciences Research Hub, Imperial College London, White City Campus, Wood Lane, W12 0BZ London, UK; 3 Department of Chemistry, University College London, Gordon Street, WC1H 0AJ London, UK; 4 Department of Life Sciences, Imperial College London, South Kensington Campus, SW7 2AZ London, UK

**Keywords:** Biophysical chemistry, Colloids, Nanoparticles

## Abstract

The rapid development of nanotechnology has led to an increase in the number and variety of engineered nanomaterials in the environment. Gold nanoparticles (AuNPs) are an example of a commonly studied nanomaterial whose highly tailorable properties have generated significant interest through a wide range of research fields. In the present work, we characterise the AuNP-lipid membrane interaction by coupling qualitative data with quantitative measurements of the enthalpy change of interaction. We investigate the interactions between citrate-stabilised AuNPs ranging from 5 to 60 nm in diameter and large unilamellar vesicles acting as a model membrane system. Our results reveal the existence of two critical AuNP diameters which determine their fate when in contact with a lipid membrane. The results provide new insights into the size dependent interaction between AuNPs and lipid bilayers which is of direct relevance to nanotoxicology and to the design of NP vectors.

## Abstract (web summary)

Citrate-stabilised gold nanoparticles are used in a range of applications, but their interaction with lipid membranes is not fully understood. Here the effect of nanoparticle size on the interaction with POPC and DOPC vesicles is studied experimentally.

## Introduction

Engineered nanomaterials (ENMs) are used in a wide range of sectors including medicine, technology, cosmetics and food and their growing use is raising serious concerns regarding their impact on human health^1,2^. In order to be able to distinguish between harmless and harmful ENMs, significant progress must be made in understanding the key initiating events at bio-nano interfaces and determining the NP properties relevant to these events. One of the key events contributing to nanotoxicity is the interaction of ENMs with phospholipid membranes. For this reason, lipid membranes such as phospholipid vesicles are commonly exploited as a minimal model system for investigating the biological impact of ENMs^3–7^. It has been demonstrated that nanoparticles can be internalised within both cells or model membranes via non-specific interactions, in the absence of receptor-mediated uptake^4,6,8^. The fate of a nanoparticle in contact with a lipid surface is determined by the elastic properties of the membrane and their balance with the adhesion forces. These energetic contributions define the degree of membrane deformation and NP wrapping^3,9–11^. The NP-membrane interaction can indeed lead to different scenarios: NP internalisation within the membrane bilayer^12^, full NP engulfment (similar to cellular non-specific endocytosis)^3^ or NP absorption on the external membrane surface^13^.

Among the many possible ENMs, spherical gold nanoparticles (AuNPs) have emerged as promising candidates for biomedical diagnostic, therapeutic and photoelectrochemical applications^14–16^ due to their facile surface chemistry, ease of synthesis and tuneable size. Depending on the specific AuNPs, membrane and dispersant properties, various interaction outcomes have been observed^5,6,12,17–19^. For example, cationic^20^ or hydrophobically^12^ functionalised AuNPs (<5 nm) can successfully be internalised within the membrane bilayer. On the other hand, anionic AuNP of the same size only interact with the outer surface of the membrane^20^. In addition, recent work also demonstrated that citrate-stabilised AuNPs (<15 nm) strongly interact with zwitterionic phosphocholine (PC) lipids and how the formation of AuNPs aggregates on liposomes surface depends on the fluidisation of the lipid membrane^21–23^ and the presence of protein corona^24^.

**Fig. 1:**
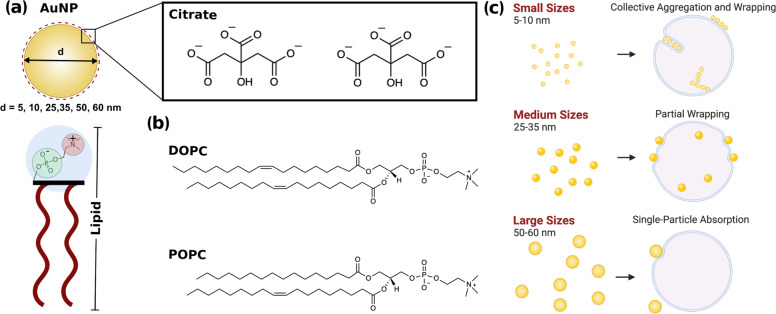
Schematic representation of the AuNP and lipid interactants and diverse outcomes arising from AuNPs–lipid membrane interactions at different size ranges. **a** Schematic of the citrate-stabilised AuNP and PC lipid interaction due to a combination of electrostatic and van der Waals interactions. Chemical structure of **b** DOPC and POPC phospholipids. **c** Summarising representation of the diverse AuNP–lipids interaction outcomes at different nanoparticles size range.

Our study provides insight into the fate of citrate-stabilised AuNPs when interacting with lipid model membranes and investigates the size dependence of this physical chemical interaction (Fig. 1a). We employ citrate-stabilised AuNPs with a diameter ranging from 5 to 60 nm (i.e., 5, 10, 25, 35, 50 and 60 nm) and two formulations of unilamellar liposome (diameter of ~180 nm): POPC (1-palmitoyl-2-oleoyl-glycero-3-phosphocholine) and DOPC (1,2-dileoyl-*sn*-glycero-3-phosphocholine). Both POPC and DOPC (lipid structure in Fig. 1b) are PC lipids with a zwitterionic head group and consequently a neutral net charge, however, they differ in the structure of their lipid tails. Our aim is to better understand the role of size in determining the behaviour of NPs in contact with a lipid membrane. We show that citrate-stabilised AuNPs in the range from 5 to 60 nm interact with large unilamellar vesicles (LUVs) very differently as schematically represented in Fig. 1c. Lipid membranes are highly flexible, and the bilayer can be deformed due to AuNP adhesion on its surface. Smaller AuNPs (5–10 nm) tend to form aggregates on the bilayer surface or are engulfed by the formation of wrapped linear aggregates within a tubular membrane. Conversely, larger AuNPs (25–35 nm) adsorb on the outer surface with an observable bending of membrane and characteristic penetration depth. For diameters between 50 and 60 nm, the AuNP-membrane absorption events are affected by the increase of the membrane tension due to the decrease of the liposome/AuNP’s surface area ratio. We also couple these observations with the quantification of the enthalpy change during the AuNP–membrane interaction.

## Results and discussion

### Theoretical background

Investigating how NPs interact with lipid membranes is fundamental to understanding their bioactivity. The fate of a nanoparticle in contact with a lipid surface is determined by the elastic properties of membrane and their balance with the adhesive forces between them. These energetic contributions define the degree of membrane deformation and wrapping of NP. The spontaneous NP wrapping is possible when the adhesion energy between the NP and membrane ($W$) is sufficiently strong to overcome the energy cost associated with the membrane bending ($k$). Spontaneous NP wrapping has been observed in both cells^25^ and model membranes such as polymersomes^26^ and liposomes^13,24,27^. Theoretical (based on elastic theory for a sphere interacting with a two dimensional sheet^10^) and experimental studies identified the existence of a critical NP diameter whose value, in the case of a tensionless membrane, is defined by the ratio between these two energetic contributions: $dc\, {\it{ \equiv }} \, 2\sqrt {2k/\left| W \right|}$^9,10^. This value defines the minimum NP diameter needed to observe the spontaneous NP’s wrapping within the lipid bilayer. For example, the critical diameter for silica NPs has been determined to be around 20–25 nm, corresponding to a few mJ m^−2^ of adhesive strength^28,29^. Note that the critical diameter changes depending on the nature of the nanoparticle surface. Molecular simulations^30,31^ have shown that for neutral crystalline silica surfaces the critical diameter as defined above, obtained from predictions of $W,$ is ~16 nm while for amorphous silica is ~10 nm. For AuNPs the critical diameter is much smaller (~4 nm) based on the experimental data/simulation prediction of $W$ = 42 mN m^−1 ^^32^ for a bare gold surface.

For larger membrane deformations, however, the membrane tension comes into play. The elastic energy of the membrane will not only depend on the bending energy but also on the tension contribution. In this case, NPs larger than the critical radius will remain partially wrapped. The depth of penetration depends on the value of the surface tension and it decreases with the increase of NP’s size. The NP full wrapping is reached only at a second critical NP diameter: $dc,\sigma \, {\it{ \equiv }} \, 2\sqrt {2k/\left| W \right| - \sigma }$, which characterises a larger NP’s limiting size.

Refinement of the elastic theory to include the finite thickness of the membrane (~4 nm) and the variation of $W$ with NP diameter^33^ leads to a new contact energy $w_{\rm{SLB}}^{\rm{s}}$ (where supported lipid bilayer (SLB) recognises that the energy may be different to a free bilayer) and more accurate critical diameters. We defined a new contact energy: $w_{\rm{SLB}}^{\rm{s}} = - k_w + \frac{m}{a} - \frac{{2\kappa _{\rm{SLB}}}}{{a^2}}$, where $- k_w$ is the simulated absorption strength on a flat surface^30–32,34^, $a$ is the wrapping diameter equal to the nominal radius plus 2 nm and $m$ and $\kappa _{\rm{SLB}}$ are adjustable constants fitted to coarse grained simulation data for $w_{\rm{SLB}}^{\rm{s}}$. This approach gives a critical diameter for adhesion for AuNPs equal to zero. So that the theory predicts AuNPs always adhere while the critical diameters for other common nanoparticles such as uncharged silica are: quartz 41 nm, cristobalite 25 nm and amorphous silica 7.5 nm. The effect of negative surface charges either raises or lowers the critical diameter depending on the surface considered^33^ and NP geometry also plays a significant role with cylindrical NPs have a critical diameter < spheres which may make it favourable for spheres to aggregate into rod-like structures^33^.

### Light-scattering analysis

In this study, we investigate the size effect of citrate-stabilised spherical AuNP on the interaction with lipid vesicles. We employed a broad size range of AuNPs: 5, 10, 25, 35, 50 and 60 nm, with two formulations of LUVs: POPC and DOPC. All the experiments were carried out with an excess of AuNPs’ surface area in orders of magnitude larger than a potential full layer coverage of lipid membrane over the gold surface. While the AuNPs concentration in solution is provided by the supplier, the maximum concentration in liposomes per millilitre has been estimated as follow by assuming no loss of phospholipid during the vesicles’ preparation and their complete unilamellarity. The average diameter of vesicles (179 nm) has been obtained from the size distribution profile of their cryo-EM micrographs (Supplementary Fig. 1a, b) and thus, the liposome concentration estimated as 7.1 × 10^9^ liposome/mL (see “Methods” for details). As expected, the average diameter of vesicles calculated from the EM micrographs is lower than the dynamic light scattering (DLS) characterisation of hydrodynamic diameter (Supplementary Fig. 1c). The order of magnitude of the liposome concentration has been validated by both multi angle DLS particle counting analysis (5.03 × 10^9^ liposome per mL) and calcein assay (2.42 × 10^9^ liposome/mL) for the detection of the total intra-vesicle volume in solution (details of experiments can be found in the “Methods” section, Supplementary Fig. 2a, b and Supplementary Note 1). To exclude the presence of AuNP’s aggregation, all sizes were characterised by UV and TEM (Supplementary Fig. 3a, b).

**Fig. 2:**
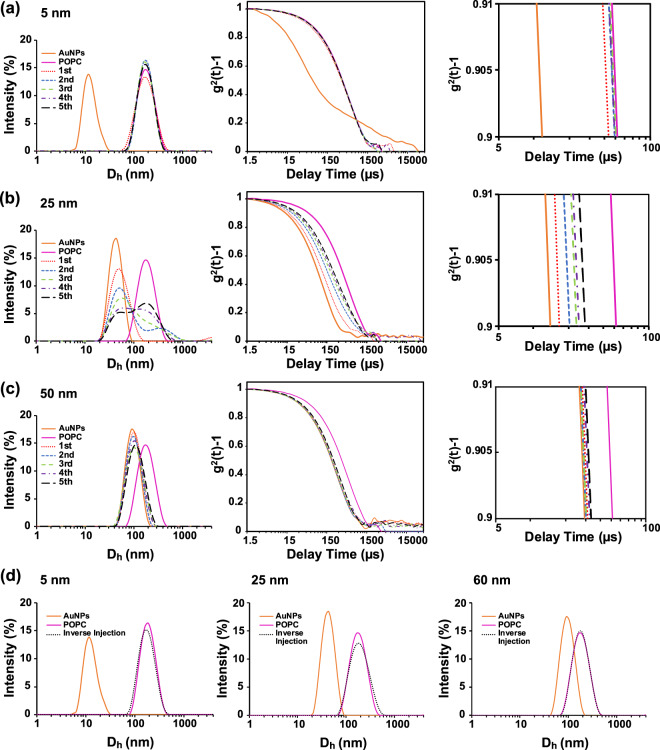
Size distribution and autocorrelation profiles of the interaction of AuNPs with LUVs. Size distribution and autocorrelation profiles of **a** 5 nm, **b** 25 nm and **c** 60 nm by injecting LUVs into the AuNPs dispersion All the profiles were obtained by subsequent injections of LUVs within an AuNPs dispersion, up to five injections (1st injection = dotted red line, 2nd injection = dashed blue line, 3rd injection = dashed green line, 4th injection = dashed violet line and 5th injection = dashed black line). Magnification of the autocorrelation profiles between 5 and 100 µs of delay times is shown on the right end side of the figure. **d** Size distribution profiles of inverse injection: smaller (10 nm), medium (25 nm) and larger (60 nm) size AuNPs into the LUVs dispersion. Control samples were obtained by measuring the AuNP (orange) and LUVs (magenta) dispersions.

The interaction between AuNPs and liposomes has firstly been investigated by DLS. The DLS size distribution profiles and autocorrelation functions for all the AuNPs/lipid formulations investigated are illustrated in Supplementary Figs. 4 and 5, respectively, while here we highlight the 5, 25 and 60 nm profiles of the POPC formulation. The DLS investigations clearly show that the AuNP–membrane interaction is strongly size dependent. For the experiment, up to five subsequent injections of LUVs into the AuNP dispersion have been made, each increasing the surface area of LUV in solution. Considering that the experiment was carried out with an excess of AuNPs (i.e., hundreds of AuNPs available per vesicle), we expect that the DLS signal will be governed by the dominantly scattering AuNPs. Interestingly, this was not the case for smaller AuNP sizes (5 and 10 nm). As shown in Fig. 2a, the peak of the AuNP–LUVs mixture is shifted from the peak at the gold diameter to that of the LUVs after the first injection. We ascribed this effect to the strong interaction occurring between the smaller AuNP and the LUVs. As soon as the AuNPs interact with the LUVs, the AuNP signal shifts to that corresponding to the LUV-peak area, indicating a full AuNP–lipid bilayer interaction.

This effect can also be seen in the corresponding autocorrelation function profiles. After the LUVs were injected into the solution of small AuNPs (5 nm), we observed a drastic shift in the autocorrelation function to the longer decay times of LUV (Fig. 2a, right hand side plot). The size distribution and autocorrelation function profiles of medium-sized AuNPs (25 nm) interacting with LUVs show instead a progressive shift to larger sizes and longer decay times at every LUV injection. We identify this effect with the presence of two interacting NP populations in solution: free AuNPs and AuNPs adsorbed by the LUVs. With every LUV injection, the population of free AuNPs progressively shrinks, while the interacting population grows. Therefore, the DLS measurement profiles are the sum of these two differently sized populations and the size distribution profile and decay times fall in an intermediate region between the only AuNP and only LUV signals. Finally, the DLS profiles of larger AuNPs (60 nm) show a decrease in the AuNP–LUV interaction. Following the sequential injections of LUVs within the AuNP dispersion, the signal stays in the area of the strongly scattering AuNPs, indicating that the AuNP–LUV interactions are negligible at this AuNP size range.

Inverse experiments have also been performed by injecting AuNPs within the LUV dispersion and inverting therefore the AuNP/LUV concentration ratio (the DLS profiles for all the lipid formulations and AuNPs’ sizes are illustrated in Supplementary Fig. 6). The resulting size distribution peak of LUV–AuNP mixtures stays unchanged in the LUV size-peak area (Fig. 2d). This indicates that the injected AuNPs (the minority population in solution) are all interacting with the LUVs due to the absence of the AuNP size peak even for larger AuNPs.

### Visualising the lipid–AuNPs interaction at the nanoscale

**Fig. 3:**
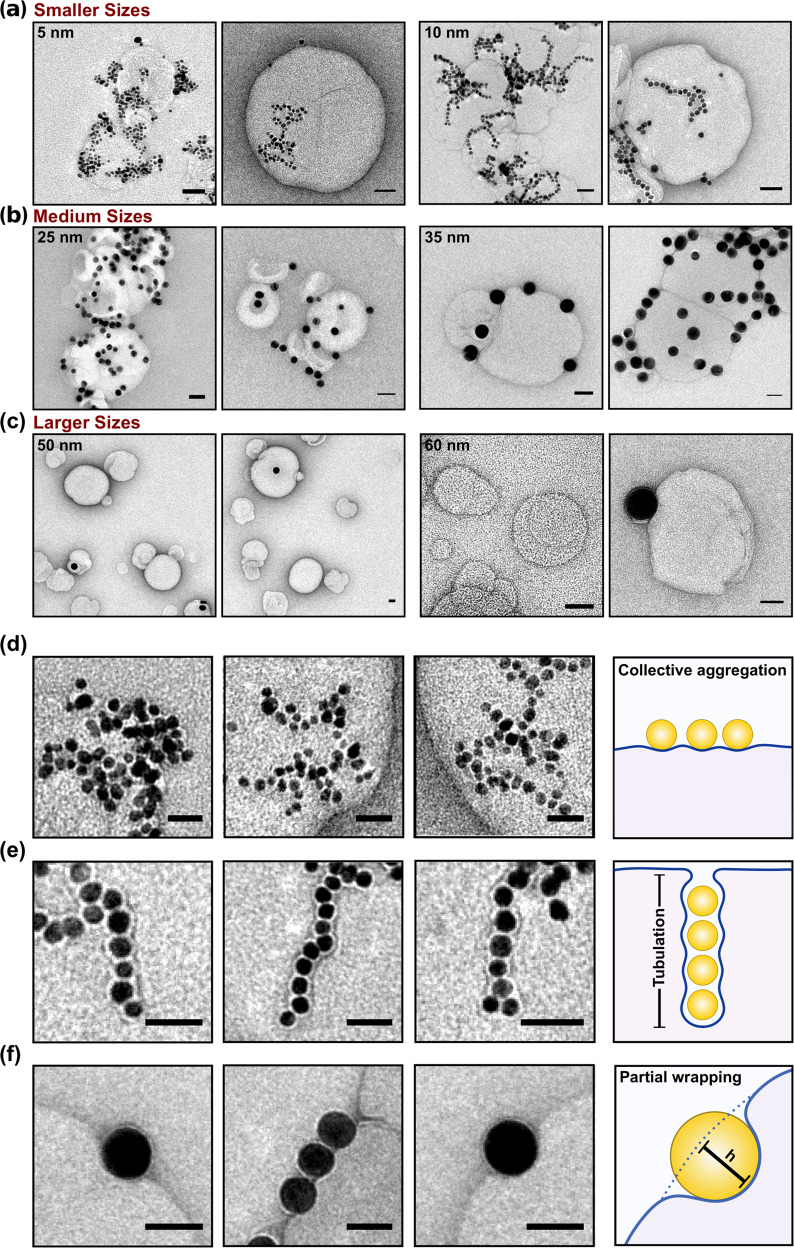
TEM micrographs of differently sized AuNPs interacting with lipid model membrane. TEM micrographs of **a** small (5–10 nm), **b** medium (25–35 nm) and **c** large AuNP (50–60 nm) interactions with membrane. Scale bar 50 nm. Depending on the *A*_v_/*A*_NP_ ratio, we observe the presence of a linear cooperative aggregation **d** longitudinal and **e** perpendicular to the bilayer and absorption plane (tubulation) or **f** absorption (partial wrapping) characterised by a measurable penetration depth. Full micrographs are show in Supplementary Fig. 7.

The AuNP–LUV mixtures characterised via DLS (Fig. 2a–c and Supplementary Figs. 4 and 5), following the first LUV injection, has been further analysed by transmission electron microscopy (TEM and cryo-EM). The micrographs relative to these investigations are shown in Fig. 3 and Supplementary Fig. 7.

For the interaction of 5 and 10 nm AuNPs with both DOPC and POPC liposomes’ membrane (Fig. 3a), we observed two correlated outcomes: AuNPs aggregation on the liposome surface, particularly evident for 5 nm AuNPs, and the formation of an inhomogeneous decoration of the lipid vesicles. For the 10 nm AuNPs, a linear organisation is observed (Fig. 3a). AuNPs aggregation on the outer surface has been previously reported for ~15 nm AuNPs interacting with PC membranes^21–23,35^. However, the previous work did not report any membrane bending deformation arising from the AuNPs interaction. This has been ascribed to the presence of an induced lipid gelation at the point of contact AuNP/lipids (in the presence of salt in the dispersant) that decreases the membrane fluidity^21,23^ or the shorter time scales of the experimental window^22^. Conversely, our results show that the small AuNPs can also be wrapped by the outer membrane layer within an invagination of tubular shape into the liposome. These tubular deformations can be both present as a cooperative aggregation on the membrane surface and internalised tubular aggregates. On the other hand, medium (25 and 35 nm) and larger (50 and 60 nm) AuNPs in Fig. 3b, c clearly sit on the outer layer of membrane with a measurable depth of penetration. Note, the presence of adsorbed AuNPs was barely detectable in both TEM and DLS investigations at 50 and 60 nm sizes (Fig. 3b, c). We discuss these different outcomes below.

When AuNPs absorb on the liposome’s membrane surface, it can respond by adjusting its volume and changing its shape. The local deformation of the membrane, which is visible in the micrographs Fig. 3, reflects the interplay between adhesion and the elastic response of the liposome. Considering the vesicle (A_v_) and the AuNP area (*A*_NP_), the *A*_v_/*A*_NP_ ratio drastically decreases with an increase in the AuNP diameter. At the employed sizes, the *A*_v_/*A*_NP_ ratio passes from ~1300 for the 5 nm and ~320 for the 10 nm AuNPs to ~51 and ~8.9 for the 25 and 60 nm respectively. This indicates that the membrane change of shape and the maximum number of internalised or adsorbed AuNPs within a 179 nm liposome decreases with the nanoparticle size. Conversely, the decrease of the A_v_/A_NP_ ratio leads to an increase of the bending energy contribution.

As noted above, accordingly to the extended elastic theory^33^, all AuNPs will adhere to liposomes and enter in the liposome lumen by endocytosis, until the increase in tension (surface and bending) shuts out further complete wetting at a maximum tension. For a 179 nm liposome only spherical NPs with diameters <15 nm can enter before lockdown is complete. For these NPs only very small (and perhaps undetectable) numbers need to enter until the liposome allows only partial wrapping. For example, only two NPs of diameter 10 nm inside the liposome (*d* = 179 nm) are sufficient to lock out further nanoparticles from entering: so, it is no surprise that we did not detect full wrapping in the images. AuNPs can still decorate the surface and indeed cooperate to distort the membrane surface. Interestingly, the micrographs of the liposome’s interaction with 10 nm AuNP show the presence of a linear cooperative wrapping longitudinal (Fig. 3d) and perpendicular to the bilayer and absorption plane in the shape of tubular structures (Fig. 3e). In the former case, the AuNPs are equally wrapped by the lipid membrane. However, in the latter case, the AuNPs appear to be enclosed within a membrane tube with a ~3 nm distance between them. The tubular structure has an average diameter of 20 nm and a length of a hundred of nanometres. Theoretical simulations^11,36^ demonstrate that cooperative wrapping and tubular deformation are energetically favourable compared to the single wrapping of nanoparticles. The energy cost of rotationally symmetric shapes of membrane tubes is in fact lower than multiple membrane segments which characterise single wrappings^36^. The micrograph in Fig. 3e clearly shows how the periodic shaping of the membrane around the wrapped AuNPs along the tubular structure.

Partial wrapping of AuNPs is observable at lower *A*_v_/*A*_NP_ ratios, i.e., larger AuNP sizes. Figure 5f shows the absorption of medium size AuNPs characterised by a measurable penetration depth of ~10 nm which is larger than the membrane thickness (~4 nm). This confirms that fluid lipid membranes are very flexible, and the perturbations induced by the nanoparticle absorption are significantly larger than their thickness.

As mentioned, previous work investigated the interaction of ~15 nm citrate-stabilised AuNPs with PC membranes^21–23^. However, no membrane perturbation has been observed at the point of contact AuNP/membrane. These studies were carried out in the presence of salt and/or buffer. The presence of salt can weaken the electrostatic interactions between NPs rendering perturbation or particle-filled membrane tubes challenging to achieve. In our case instead, in the absence of salt, the local deformation of the membrane is clearly visible in the micrographs and it is a confirmation of the different elastic interaction interplay between the NPs and lipid membrane.

Membrane deformations are only possible when NPs strongly adhere with membranes. The strength of the interaction was confirmed by centrifuging the AuNP–LUV mixtures and analysing them under cryo-EM. For this experiment up to five subsequent injections of LUVs into 5, 25 and 60 nm diameter AuNP dispersions were made as described for the DLS experiment. In order to reach a sample concentration suitable for the cryo-EM characterisation, the mixtures were concentrated under gentle centrifugation at 6 × 1000 rcf to obtain LUV deposition. This centrifugation speed separates free liposomes and free AuNPs contained in the supernatant, concentrating the LUVs/AuNP structures in the resulting pellet (Supplementary Fig. 8). The greatest sedimentation was observed in the small and medium AuNPs’ mixture (i.e., 5 and 25 nm), which exhibited a visible red pellet indicating the presence of LUVs/AuNP complex at the bottom of the Eppendorf where the LUVs are concentrated (Supplementary Fig. 8). In the case of larger AuNPs–LUVs interaction, little sedimentation was observed and most of the sample stays dispersed in solution indicating the low AuNPs–LUVs conjugate content—further indicating the attenuated interaction between the membrane and the large AuNP sizes relative to smaller particles. It follows that the bending cost decreases and the adhesive interaction increases passing from 60 to 5 nm.

**Fig. 4:**
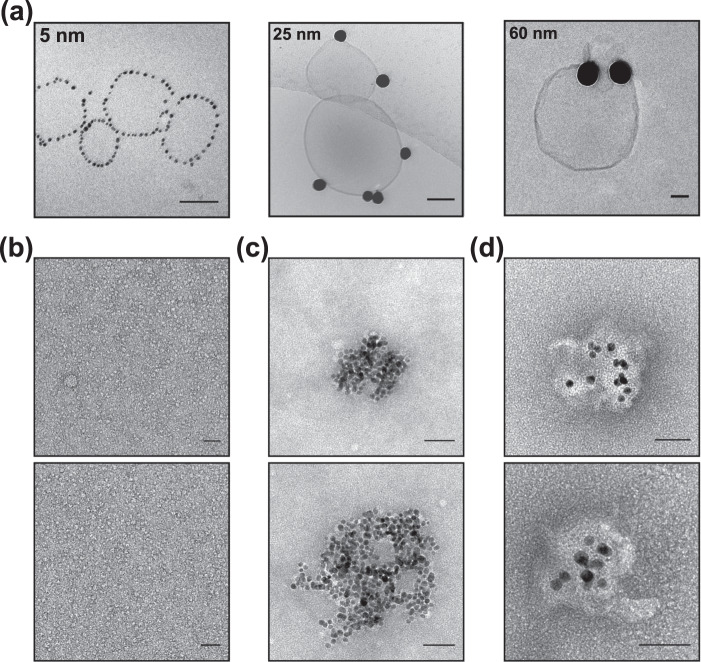
EM micrographs of differently sized AuNPs interacting with lipid model membrane and micelles. **a** The AuNPs–LUVs mixture of 5, 25 and 60 AuNP interacting with LUVs, has been concentrated by gentle centrifugation and imaged by Cryo-EM. TEM micrographs of **b** DHPC micelles interacting with **c** 5 nm and **d** 10 nm AuNPs. Scale bar 50 nm.

Cryo-EM micrographs of the resuspended pellets are shown in Fig. 4a. Smaller NPs were evenly and linearly distributed around the liposomes, confirming the previous TEM observations. Medium size AuNPs are instead randomly adsorbed on the LUV’s surface. Free AuNPs and AuNPs interacting with LUV are observed in the sample made with larger sizes. The absence of tubular deformations can be ascribed to the altered number of interacting AuNPs due to the centrifugation step.

The tubular engulfment events were visible only in the 10 nm AuNPs’ characterisation. For this reason, the coordination of smaller size AuNPs (i.e., 5 and 10 nm) in the presence of lipids, has been further investigated by mixing them with DHPC micelles. In the presence of dispersed micelles in solution (Fig. 4b), lipids and AuNPs cluster together forming AuNP-lipids aggregates for both 5 and 10 nm AuNPs (Fig. 4c, d). However, the morphologies of the two AuNP-lipid aggregates are different: the distance between AuNPs and between AuNPs and edge of the aggregate is larger for the 10 nm AuNPs than for the 5 nm AuNPs. This effect can be related only to the difference in surface curvature of the two AuNP populations. Having a larger surface area, 10 nm AuNPs can interact and coordinate with a larger number of micelles, increasing the distance between NPs in the aggregate relative to 5 nm AuNPs.

### Nile Red fluorescence quenching

**Fig. 5:**
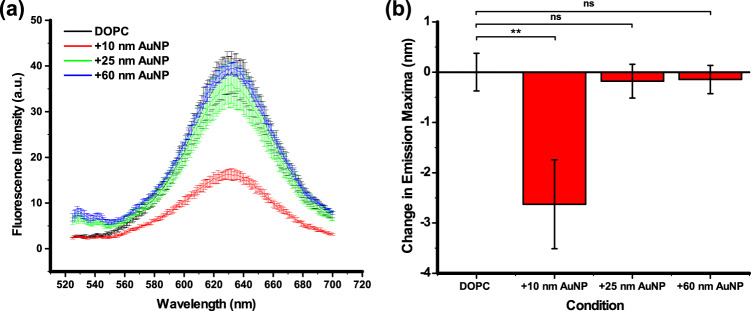
Change in Nile Red emission maxima as a function of AuNPs interaction with membrane. **a** A reduction in Nile Red fluorescence intensity is only observed upon addition of 10 nm AuNP. **b** A change in the emission maxima of the solvatochromic dye Nile Red shows a size-dependent interaction of gold nanoparticles with lipid membranes.

Further investigation on the AuNP positioning with respect to the membrane has been carried out through spectroscopic measurements of the environment-sensitive probe Nile Red (NR) shown in Fig. 5. If dispersed in aqueous solution, the fluorescence of NR is quenched by water as this probe is sensitive to polarity^37^ but the probe emits fluorescence when is embedded in the tail region of the lipid membrane. In addition, NR can also be quenched, and therefore lose its inherent fluorescent properties, when in close proximity with metallic nanoparticles, through fluorescence resonance energy transfer^38^. Furthermore, the NR emission maxima changes as a function of solution polarity in a way that a blue shift in emission is attributed to a decrease in environment polarity, whilst, a red shift in emission is attributed to an increase in environment polarity^39^. Literature reports that 40 mol% addition of cholesterol to lipid vesicles resulted in an 11 nm blue shift in NR emission maxima^40^. This is attributed to NR residing deeper in the membrane (as confirmed via the parallax method where a change in NR fluorescence occurs upon interaction with fluorescence quenchers located at different membrane depths) when cholesterol is present in the membrane. So, an alteration of the solvent environment and fluorescence intensities of NR is an indication of the AuNP interaction at the head group/tail interface. The AuNP interaction with the bilayer interface as a function of their size has been carried out employing 10, 25 and 60 nm diameter nanoparticles. As can be seen from Fig. 5a, there is a sequential reduction of NR fluorescence intensity which reflect the decrease of the *A*_v_/*A*_NP_ ratio with the increase of the AuNP diameter and consequently different AuNPs mode-of-action. In the absence of AuNPs, the NR fluorescence is at its maximum. It undergoes small changes with the addition of 60 nm and continues to decrease with 25 nm AuNPs. A fourfold reduction in fluorescence intensity is instead observed upon the addition of 10 nm NP (Fig. 5b). The literature reports a similar intensity drop to the quenching effect of AuNPs when in the close proximity with this probe^38^ and upon the addition of cholesterol to DOPC vesicles^40^, attributed to increased water penetration in the interfacial region of the membrane (where NR is located). It is expected that membrane remodelling upon introduction of small nanoparticles would result in increased contact with NR and also water penetration due to the volume remodelling.

AuNPs can act as fluorescence quenchers where the fluorophore acts as the donor and the NP/quencher acts as the acceptor. This energy transfer is dependent on the distance between these two compounds in a way that it occurs when their distance is short. On the other hand, when the donor and acceptor are distant, fluorescence occurs. Due to the decrease of the *A*_v_/*A*_NP_ ratio, the absorption of larger AuNPs (60 nm) is inhibited by the increase energy cost of bending and therefore, the absorption events reduced. This means that the majority of the larger AuNPs stay free in solution instead of attached to the membrane surface as indicated from the DLS and TEM analysis above leading to a small change in the NR fluorescence intensity. In conclusion, the combination of fluorescence reduction in more polar solvents and AuNP quenching effect for NR induces this intensity change, which further indicates the size dependence of the interaction between AuNPs and liposomes.

Interestingly, only the addition of 10 nm AuNPs also resulted in a blue shift indicating a decrease in membrane polarity (Fig. 5b). Larger AuNPs (25 and 60 nm), however, gave a negligible shift in fluorescence emission maxima, which is within experimental noise as indication of their peripheral interaction with the outer surface of the membrane.

### Fluorescence leakage assay

Membrane disruption is one of the potential effects associated with nanotoxicity. For this reason, a fluorescence leakage assay, using calcein as a fluorescence dye, was carried out to assess the degree of membrane disruption. As a control experiment, the potential quenching effect of AuNPs has been investigated by recording the calcein fluorescence (at the same concentration of AuNPs and calcein used in the leakage assay) in the presence of AuNPs and absence of LUVs. These control experiments for all the AuNP sizes indicate that the maximum calcein fluorescence stays unchanged in the presence of AuNPs (Supplementary Fig. 9a, b).

**Fig. 6:**
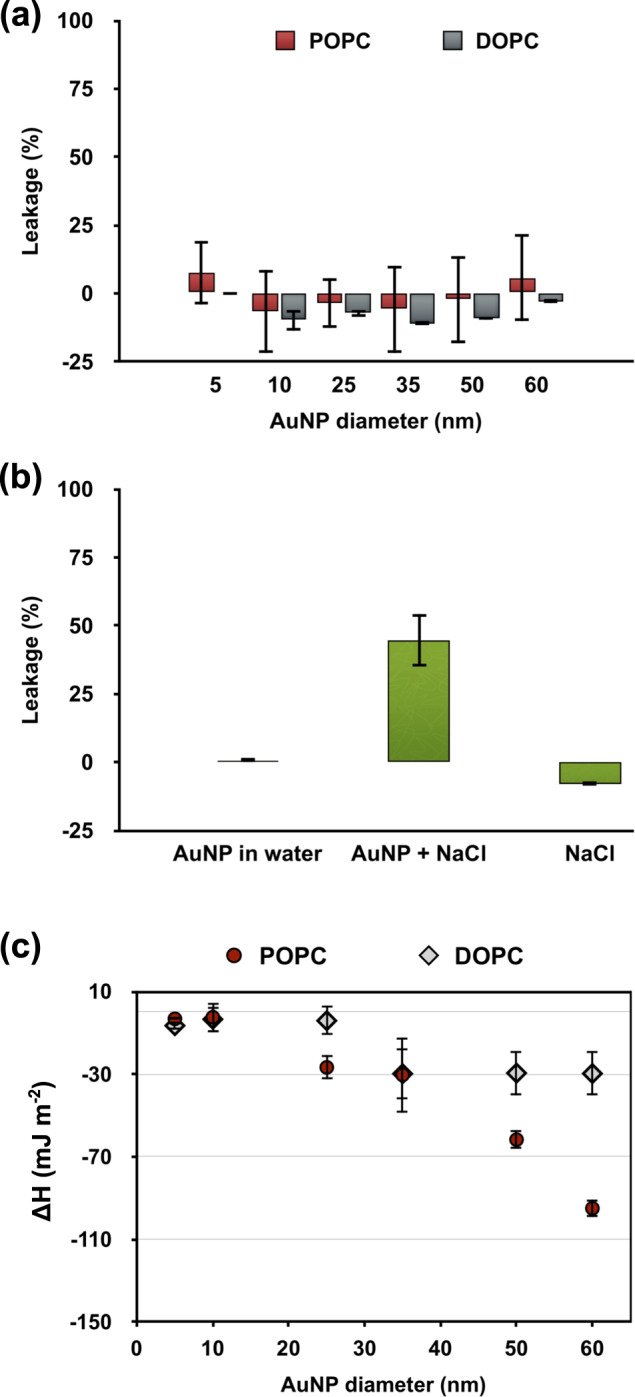
AuNP–LUV quantification of interaction and membrane leakage assay results. **a** The membrane leakage assay has been performed at the same conditions as all the data in order to detect any membrane disruption consequent to the interaction with AuNPs. **b** Summary of the LUVs leakage assay under different conditions: in the presence of 10 nm AuNPs in water and NaCl, and in the presence of salt and absence of AuNPs. **c** Enthalpy changes for the POPC (red) and DOPC (grey) formulations versus the AuNPs sizes. The error bars represent the standard error calculated across the range of samples.

For the leakage assay, calcein dye was encapsulated at a self-quenching concentration of 50 mM into vesicles and the sample purified via size exclusion chromatography from the free dye in solution. Liposome leakage is therefore detected as a fluorescence increase. In order to balance the osmotic pressure across the liposome membrane due to the calcein encapsulation, the liposomes were dispersed in 500 mM sucrose solution. Figure 6a shows the percentage of fluorescent dye leakage as a function of the AuNP diameter. For all the formulations investigated, the membrane leakage is negligible, and thus the assay was negative. To further investigate the possibility of interference from sucrose molecules on the AuNP/liposome interaction, the leakage assay has been repeated in the absence of sucrose (Supplementary Fig. 9c). The assay resulted in a negligible leakage and therefore the sucrose has no effect on the membrane–AuNP interaction.

We also investigated the effect of salt on the AuNPs/liposome interaction recording the fluorescence change over time. As stated before, previous studies reported membrane leakage using a similar system (i.e. ~15 nm AuNP and ~100 nm liposomes) in the presence of salt buffer^21,23^. The authors used a 100 mM NaCl solution to disperse the liposomes and balance the osmotic pressure across the membrane due to the presence of calcein (500 mM). We reproduced this previously reported leakage in the presence of salt with our system and observed rapid calcein leakage that only occurred in the presence of both salt and AuNP, whilst AuNP interaction alone again resulted in negligible leakage (summary in Fig. 6b, kinetic of calcein release are shown in Supplementary Fig. 10). Interestingly, the dye leakage was present using the AuNPs in combination with NaCl salt only (Fig. 6b). This can be ascribed to the effect of sodium chloride on the lipid bilayer. The salt ions reduce the lipid diffusion and mobility by forming tight ion–lipid complexes^41^ and can lead also to the phase separation of PC membranes^42^. The addition of AuNPs causes a further transient perturbation or defects of the membrane, which leads to the dye leakage. This salt effect on lipid membrane is also the reason why membrane perturbation has not been detected at the point of contact AuNP/lipid in previous studies. The salt increases the rigidity of membrane^41,42^ and the AuNP remains therefore adsorbed on the surface without penetration of the membrane. These last experiments confirm that the interaction outcomes depend not only on the specific of interacting nanoparticles and membrane but also on solution properties where the interaction take place.

### Quantification of enthalpy changes of interaction

The enthalpy changes of interaction between AuNPs and liposomes have been quantified using isothermal titration calorimetry (ITC) at the same concentration employed in the previous analysis. As previously stated, the experiments were carried out in an excess of AuNPs surface area in order to guarantee detectable signals of heat changes. ITC is generally used to detect the temperature change during an interaction between reactive biomolecules (e.g., enzyme–substrate, protein–ligand, protein–ion) and it has been recently exploited for the detection of interactions between NPs and biomolecules such as proteins and amino acids^43,44^. Here, we exploit ITC as a powerful and highly sensitive technique to estimate the strength of the AuNP–LUVs interaction. In the experiment, the LUVs were titrated against the AuNPs dispersion (original heat flows are shown in Supplementary Fig. 11). In a typical enzyme–substrate ITC experiment, the recorded ∆*Q* of the first injection is normalised by the moles of the titrant component. However, in the case of titrations and interaction between NPs, it is more appropriated considering instead the interacting surface area: ∆*Q*/SA_L_ (SA_L_ = total surface area of liposomes). Moreover, a concentration of 0.05 mg mL^−1^ AuNPs has been used for the experiments over the different employed sizes. The total AuNPs surface area (SA_Au_) increases with the decrease of size. Thus, we further normalised the ∆*Q*/SA_L_ value for the ratio between total surface area of AuNP and liposomes: SA_Au_/SA_L_. Finally, to quantify the heat response due to the AuNP–LUV interaction and compare it in the size range 5–60 nm, the ∆*Q* of interaction has been normalised to the AuNPs surface area to obtain ∆*H* (mJ m^−2^). The ITC results illustrated in Fig. 6c show that the interaction of AuNPs with LUVs is exothermic for all AuNP sizes. The interaction with the 5 and 10 nm AuNPs results in a smaller enthalpy change than the larger NPs, which confirms that size determines how NPs interact with the liposome membranes. Note that the data reported here are enthalpies not free energies. To obtain the adhesion free energy of adsorption, we require the entropy change which could be obtained, for example, from simulation^32^. While smaller AuNPs undergo cooperative aggregation and wrapping, larger-sized AuNPs instead sit on the outer surface of the membrane only. This small difference in interaction between smaller and larger sizes is therefore detectable using ITC. When an AuNP adsorbs on the liposome membrane, the vesicle changes its volume and water molecules cross the membrane from the inside to the outside of the vesicle. This shape change and water molecules motion causes an increase of the entropic disorder of the system. The adhesion free energy of adsorption (∆*G*) is given by the enthalpy (∆*H*) and entropy (∆*S*) of adhesion: ∆*G* = ∆*H* – *T*∆*S*. Since the experiments are carried out in an excess of AuNPs and therefore all the lipids are potentially involved in the interaction, we can consider the same adhesion free energy of adsorption (∆*G*) for the AuNPs at all sizes. So, an increase of the entropic contribution is correlated to a decrease of the enthalpy. In the case of smaller AuNPs (≤10 nm), their aggregation and cooperative wrapping causes a large increase of the disorder of the system associated with an increase of membrane and water motion. The entropic contribution, however, decreases at lower *A*_v_/*A*_NP_ ratios for medium and larger sizes, which is reflected in an increase of enthalpy change.

Moreover, for larger size AuNPs, the ITC detected a pronounced (approximately a threefold decrease in enthalpy) difference between POPC and DOPC LUVs. POPC creates more ordered membrane structures^45,46^ compared with DOPC, which results in an increase in the membrane bending rigidity for POPC membranes due to the difference in the degree of saturation of the lipid tail and chain length. These data demonstrate the sensitivity of the ITC technique to detect the influence of membrane composition on the interaction with inorganic nanoparticles. Membrane composition may indeed have serious implications to the susceptibility of different tissues/cells types to nanotoxicity.

**Fig. 7:**
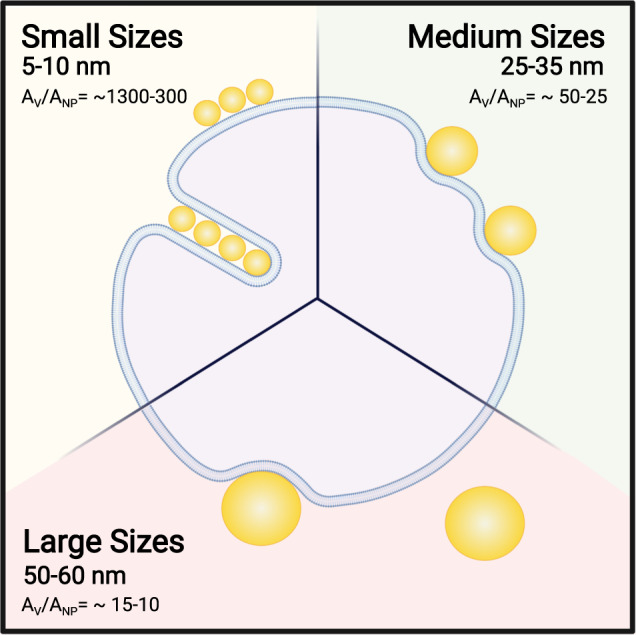
Schematic representation of the diverse outcomes arising from AuNPs–lipid membrane interactions at different size ranges. Changing the AuNP size from 5 to 60 nm, we observed three different types of outcomes: Small AuNP (5–10 nm) undergoes cooperative aggregation and wrapping characterised by the formation of a membrane tube; Medium AuNPs (25–35 nm) absorb on the membrane’s outer surface with a measurable penetration depth; Large AuNP (50–60 nm) interaction is characterised by a few absorption events due to the increase of bending cost.

In summary, we show that citrate-stabilised AuNPs in the range from 5 to 60 nm interact with LUVs very differently (Fig. 7). The vesicle (*A*_v_) and the AuNP area (*A*_NP_) ratio (*A*_v_/*A*_NP_) rules the outcomes of this interaction. Smaller AuNPs (5–10 nm) show a cooperative absorption on the outer surface of the membrane and wrapping events are also present in this size range. Larger AuNPs (25–35 nm) undergo absorption events characterised by membrane penetration depth, whereas for diameters between 50 and 60 nm the AuNP-membrane interaction is minor, characterised by few absorption events.

Investigating the physicochemical mechanisms of interaction between NPs and liposomes as a model membrane system is important for the understanding of key initiating events of nanotoxicity at the membrane interface. AuNPs are a promising ENMs exploited for different applications due to their tuneable physical and optical properties. In this study, we employed a minimal model system to investigate the AuNP–membrane interaction as a function of the AuNP size. We investigated the interaction of a wide range of AuNP size from 5 nm up to 60 nm in diameter, with lipid vesicles as a model membrane. This model system has been chosen to mimic the interaction between nanoscale AuNPs and the surface of biological membranes. In all the experiments, we find that AuNPs interact differently with LUVs according to their diameter and thus vesicle (*A*_v_) and AuNP area (*A*_NP_) ratio (*A*_v_/*A*_NP_). The experiments revealed the presence of two AuNP critical diameters at around 10 and 50 nm. At a diameter equal or below 10 nm, AuNPs undergoes cooperative absorption on the outer surface of the membrane. Particularly for the liposome’s interaction with 10 nm AuNP, a linear and cooperative wrapping is present perpendicularly to the bilayer absorption plane where the AuNPs appears to be enclosed within a tubular membrane structure. Increasing the diameter size up to 50 nm, the AuNPs are adsorbed on the LUV outer surface and induce bending of the membrane with a measurable depth of penetration. Above 50 nm, the *A*_v_/*A*_NP_ ratio is much lower and the frequency of membrane bending and absorption is greatly reduced. The increase in membrane tension due to contact with larger NPs is the cause of the consequent inhibition of wrapping as confirmed by the elastic theory, which defines a larger critical diameter when in the presence of surface tension.

Our findings offer a deeper understanding of the AuNP–membrane interaction through a systematic study of NP size effect controlling the NP-membrane interaction. These findings should be useful to better understand the key initiating events in the development of nanotoxicity as well as design of nanoparticle vectors for drug delivery.

## Methods

Chemicals were purchased by Sigma-Aldrich and used as received unless otherwise indicated.

The spherical citrate-stabilised AuNPs were purchase from Nanopartz (Loveland, CO, USA) at a gold concentration of 0.05 mg mL^−1^ and citrate concentration of 3 mM. The AuNPs mL^−1^ concentrations for the employed sizes are provided by the company: 4.76 × 10^13^ (5 nm), 6.40 × 10^12^ (10 nm), 3.72 × 10^11^ (25 nm), 1.16 × 10^11^ (35 nm), 3.97 × 10^10^ (50 nm) and 2.30 × 10^10^ (60 nm).

### Production of LUVs

The employed lipids were DOPC and POPC from Avanti Polar Lipids (Alabaster, AL, USA). Lipid films were prepared by dissolving the lipid (13.6 µmol) in chloroform, before gently vortexing the solution for 1 min. The solution was then evaporated under a stream of nitrogen to give a thin-lipid film, which was dried under vacuum overnight at room temperature. The dried lipid films were hydrated in a 3 mM citrate solution. To obtain unilamellar vesicles, the lipid suspension was freeze-thawed six times. Each freeze-thaw cycle consisted of freezing the sample in liquid nitrogen before thawing with a heat gun and vortexing for 60 s at room temperature. Samples were then extruded 21 times through a 0.2 µm polycarbonate filter (mini extruder from Avanti Polar Lipids, Alabaster, AL, USA) to yield unilamellar vesicles ~200 nm in diameter. Size distribution have been characterised by DLS, MADLS and EM analysis.

### Theoretical estimation of liposome concentration

The liposome concentration was theoretically estimated as follows. First, the total number of lipid molecules per vesicle were calculated using the equation:

1 $$N_{\rm{tot}} = 4\pi \frac{{\left( {\frac{d}{2}} \right)^2 + \left( {\frac{d}{2} - h} \right)^2}}{{a_{\rm{lipid}}}},$$

where *d* is the average diameter of vesicles (179 nm) obtained from the size distribution profile analysed from the cryo-EM micrographs (Supplementary Fig. 1b), *h* is the thickness of the lipid bilayer measured from the TEM micrographs (4 nm^47,48^), $a_{\rm{lipid}}$ is the surface area per lipid molecule, which for DOPC/POPC is 71 Å^49^. The number of liposomes per mL ($N_{\rm{lip}}$) was finally calculated by using the equation:

2 $$N_{\rm{lip}} = \frac{{M_{\rm{lipid}}\,\times\,NA}}{{N_{\rm{tot}}\,\times\,1000}},$$

where *M*_lipid_ is the molar concentration of lipid and *NA* is the Avogadro number. For a 2.5 mg mL^−1^ lipid concentration, used in this study, gives a liposome concentration of liposomes per millilitre of 7.18 × 10^9^.

### Vesicle concentration by fluorescence calcein assay

Lipid films were hydrated in calcein buffer (50 mM calcein, 3 mM citrate pH 6.5) to a concentration of 2.5 mg mL^−1^ and LUVs prepared as indicated above. The unencapsulated calcein was removed through size exclusion chromatography using a Sephadex G-50 column eluting in sucrose buffer (500 mM sucrose, 3 mM citrate) and the sample collected in a 96-well plate. The calcein fluorescence emission was detected at λ_ex/em_ = 494/514 nm on a Cary Eclipse Fluorometer (Agilent Technologies, USA) after the addition of 10 (v/v)% Triton X-100 to lyse vesicles. By referring to a calibration curve (Supplementary Fig. 2b), the total amount of calcein has been quantified. The particle concentration has been then calculated by dividing the obtained total amount of calcein with the theoretical amount of calcein encapsulated into one vesicle by considering the average diameter of vesicles (i.e., 179 − 8 nm of total membrane thickness) obtained from the size distribution profile analysed from the cryo-EM micrographs (Supplementary Fig. 1b).

### UV−Vis characterisation of AuNPs

All UV-visible spectroscopy measurements were performed on a Perkin Elmer Lambda 950 instrument with a measurement interval on 1 nm. Samples were measured in quartz cuvettes with a 1 cm path length. UV–Vis absorption spectroscopy is a common analytical tool used to monitor AuNP aggregation and this is done by monitoring their surface plasmon resonance (SPR) peak and any colour changes. SPR refers to the collective oscillation of electrons on the surface of gold after photon excitation and results in an absorption peak in the sample’s visible spectrum. A shift to longer wavelength of the absorption peak is observed as the diameter of AuNPs increases due to dynamic depolarisation^50^. If aggregates exist, the UV–Vis spectra will differ significantly from that of a dispersed colloid (i.e., well dispersed AuNPs). Large aggregates induce a lowering of the UV extinction, and this increases with aggregate size. Smaller aggregates typically result in a shift and broadening of the SPR peak. This is because peak broadening or shifting of the SPR speak in the UV–Vis is an expression of the aggregate’s response to visible light and these phenomena are well established in the literature^51–53^. Figure 2 in Supplementary Information shows the characteristic and progressive plasmon band red shift as the diameter of AuNPs increases with no reduction in UV extinction or unusual peak broadening or shifting, which confirms the absence of AuNPs aggregation. In addition, since AuNPs are known to aggregate when stored for several months, fresh solutions of AuNPs were always carefully characterised before use in all experiments described in this work.

### Dynamic light scattering

The samples were analysed using a Malvern Panalytical DLS instrument. The sample was crossed by a 120-mW He–Ne laser at 630 nm at a controlled temperature of 25 °C. The scattered light was measured at an angle of 173°. For the analysis, the samples were prepared at the same concentration/stoichiometry used and described in the ITC paragraph. Since the ITC technique is automated for injection and in order to allow the analysis of the AuNP/liposome mixture after every single injection, the DLS experiment was carried out in the same way by manually preparing the equivalent mixtures. Then, each of them has been diluted with a 3 mM citrate solution at a final concentration of AuNPs of 1 µg mL^−1^ into a final volume of 500 µl. The samples were analysed into a polystyrene cuvette (Malvern Panalytical, DTS0012).

The multi-angle dynamic light scattering (MADLS) analysis of the vesicle suspension was carried out using a Malvern Panalytical Zetasizer Ultra. The scattered light was measured by combining scattering information from multiple angles (backscatter, side scatter and forward scatter). For the analysis, the sample has been prepared to a final volume of 1 mL. The particle concentration distribution has been obtained from the MADLS analysis using the Malvern Panalytical Zetasizer Ultra. The particle concentration measurement process is fully automated and obtained from the Malvern Panalytical Zetasizer Ultra. Briefly, the instrument records the time averaged photon count-rate scattered by the sample to which is subtracted the background contribution from the dispersant (3 mM citrate) obtained in an individual and separated recording. Then, the instrument uses the toluene reference count rate to calculate the differential scattering cross-section for each particle size population and consequently, the absolute particle concentration distribution.

All the DLS data were processed using a Dispersion Technology Software (Malvern Panalytical) and the size distribution are shown considering the intensity-weighted particle size distribution.

### Isothermal titration calorimetry

ITC was performed by using the automated and high-sensitive MicroCal PEAQ-ITC from Malvern Panalytical. LUV and AuNP solutions were equilibrated to room temperature and degassed prior titration. The instrument’s cell was equilibrated for at least 30 min at a temperature of 25 °C prior use and maintained at the same temperature during the experiment. The heat changes were recorded as the differential power applied to maintain zero-temperature difference between the sample and reference cell, filled with the sample media (3 mM citrate). A first 0.4 µl injection was followed by a total of 12 consecutive injections of 3 µl each containing LUVs at a lipid concentration of 2.5 mg mL^−1^, were injected into cell (205 µl) filled with the employed AuNPs (0.05 mg mL^−1^). Injections were made at 150 s intervals and at 0.5 µl s^−1^ rate. A constant stirring speed of 750 rpm was maintained during the experiments to ensure sufficient mixing after each injection. For calculation of the enthalpy of AuNP–membrane interaction, the heat of dilution was measured in separate liposomes into citrate solution titrations and was subtracted. The obtained value of heat change (∆*Q*) from the first LUVs injection, which represent the 100% interaction, has been then normalised by dividing them for the total surface area of AuNPs and therefore transformed in ∆*H*, mJ m^−1^. All the data were made in triplicates.

### Transmission electron microscopy

A 2% uranyl acetate (UA) solution was used as a negative staining agent. Five microlitre of AuNP/LUVs dispersion was deposited onto glow-discharged 200 square mesh copper grids (Agar scientific). After 1 min, the grids were blotted with filter paper and then immerse into the UA staining solution for 20 s. Then, the grids were blotted again. Grids were imaged using a JEOL JEM-2100F fitted with a Gatan Orius SC 1000 camera (2 × 4k).

### Cryogenic transmission electron microscopy

Samples were vitrified using a Vitrobot Mark IV (FEI) system under controlled temperature (21 °C) and humidity (100%). Four microlitre of sample was deposited on Quantifoil copper grids with 2 µm holey-carbon on 200 square mesh (Agar scientific) and vitrified by plunging the grid into liquid ethane and transferred to liquid nitrogen. The grid is then quickly placed in a cryogenic stage and kept at −180 °C. Micrographs were collected using a Gatan 626 cryogenic holder on a FEI Tecnai 12 twin TEM operating at 120 kV with a TVIPS F216 CCD camera. The AuNP–LUV mixtures were concentrated by centrifugation at 6000 × g for 10 min, followed by removal of the supernatant and re-suspended in 10 µl 3 mM citrate solution prior the vitrification.

### Fluorescence vesicle leakage assay

Lipid films were hydrated in calcein buffer (50 mM calcein, 3 mM citrate pH 6.5) to a concentration of 10 mg mL^−1^ and LUVs prepared as indicated above. The unencapsulated calcein was removed through size exclusion chromatography using a Sephadex G-50 column eluting in sucrose (500 mM sucrose, 3 mM citrate). Calcein leakage was assessed by fluorescence spectroscopy, with the calcein fluorescence emission recorded in 96-well plates at λ_ex/em_ = 494/514 nm on a Cary Eclipse Fluorometer (Agilent Technologies, USA) on triplicate wells to obtain average fluorescent emission measurements for each vesicle composition. For all measurements, the fluorescence of a nanoparticle-free sample was used as a control to compare with AuNP-vesicle samples. The assay was firstly carried out at the employed concentration of the ITC measurements. Then, the calcein leakage was assayed to measure the effect of AuNP and vesicle concentrations change. For this purpose, a lipid concentration of 250 µM was investigated over an increased nanoparticle amounts varying from 15 to 5 µg and a constant amount of 5 µg AuNP was investigated over a lipid concentration change from 150 to 75 µM. After measurements, 10 (v/v)% Triton X-100 was added to each solution to lyse vesicles and determine the maximum fluorescence intensity for each well. Vesicles were left for 30 min to ensure complete lysis had occurred before recording final fluorescence readings. The measurement data were analysed by subtracting the control sample (vesicle solution without AuNPs) from the fluorescence intensities and normalising the result to the maximum fluorescence intensity.

### Spectroscopic measurements of the environment-sensitive probe NR

DOPC films (5 mg) containing 6 mol% NR were prepared via mixing of components in chloroform before solvent evaporation and drying of film overnight at RT in desiccator overnight. The lipid films were hydrated with 3 mM citrate buffer to a working concentration of 10 mg mL^−1^, freeze-thawed six times to ensure membrane homogeneity and extruded 21 times through a 200 nm filter. Any unencapsulated NR was removed through passage of extruded vesicles through a Sephadex G-50 column hydrated with 3 mM citrate, giving purified NR vesicles ~5 mg mL^−1^ concentration. Spectroscopy conditions: λ_ex_ = 476 nm, λ_em_ = 490–700 nm. PMT voltage = 800 V, scan rate = 120 nm min^−1^, data interval = 1 nm, average time = 0.5 s and total volume 200 µl. Data were plotted in OriginPro9. Applied standard Gaussian fit to each well to generate fit. All fits converged. Centre of peak taken as the emission maxima.

## Supplementary information

Supplementary Information

## Data Availability

The authors declare that the data supporting the findings of this study are available within the paper.
