# Supplementary material for: Size dependency of gold nanoparticles interacting with model membranes
Source: Commun Chem. 2020 Sep 17;3:130. doi: 10.1038/s42004-020-00377-y (PMC7610534; doi:10.1038/s42004-020-00377-y)
Supplement: Supplementary file 1 — Supplementary Information [file 42004_2020_377_MOESM1_ESM.docx]

**Size Dependency of Gold Nanoparticles Interacting with Model Membranes**

**Supplementary Information**

Claudia Contini^1*^, James W. Hindley^1,2^, Thomas J. Macdonald^1,3^, Joseph D. Barritt^4^, Oscar Ces^1,2^, Nick Quirke^1*^

^1^Department of Chemistry, Molecular Science Research Hub, Imperial College London, White City Campus, Wood Lane, W12 0BZ, London, UK

^2^Institute of Chemical Biology, Molecular Science Research Hub, Imperial College London, White City Campus, Wood Lane, W12 0BZ, London, UK

^3^Department of Chemistry, University College London, Gordon street, WC1H 0AJ, London, UK

^4^Department of Life Sciences, Imperial College London, South Kensington Campus, SW7 2AZ, London, UK

^*^Corresponding authors: [c.contini@imperial.ac.uk](mailto:c.contini@imperial.ac.uk) and [n.quirke@imperial.ac.uk](mailto:n.quirke@imperial.ac.uk)

**
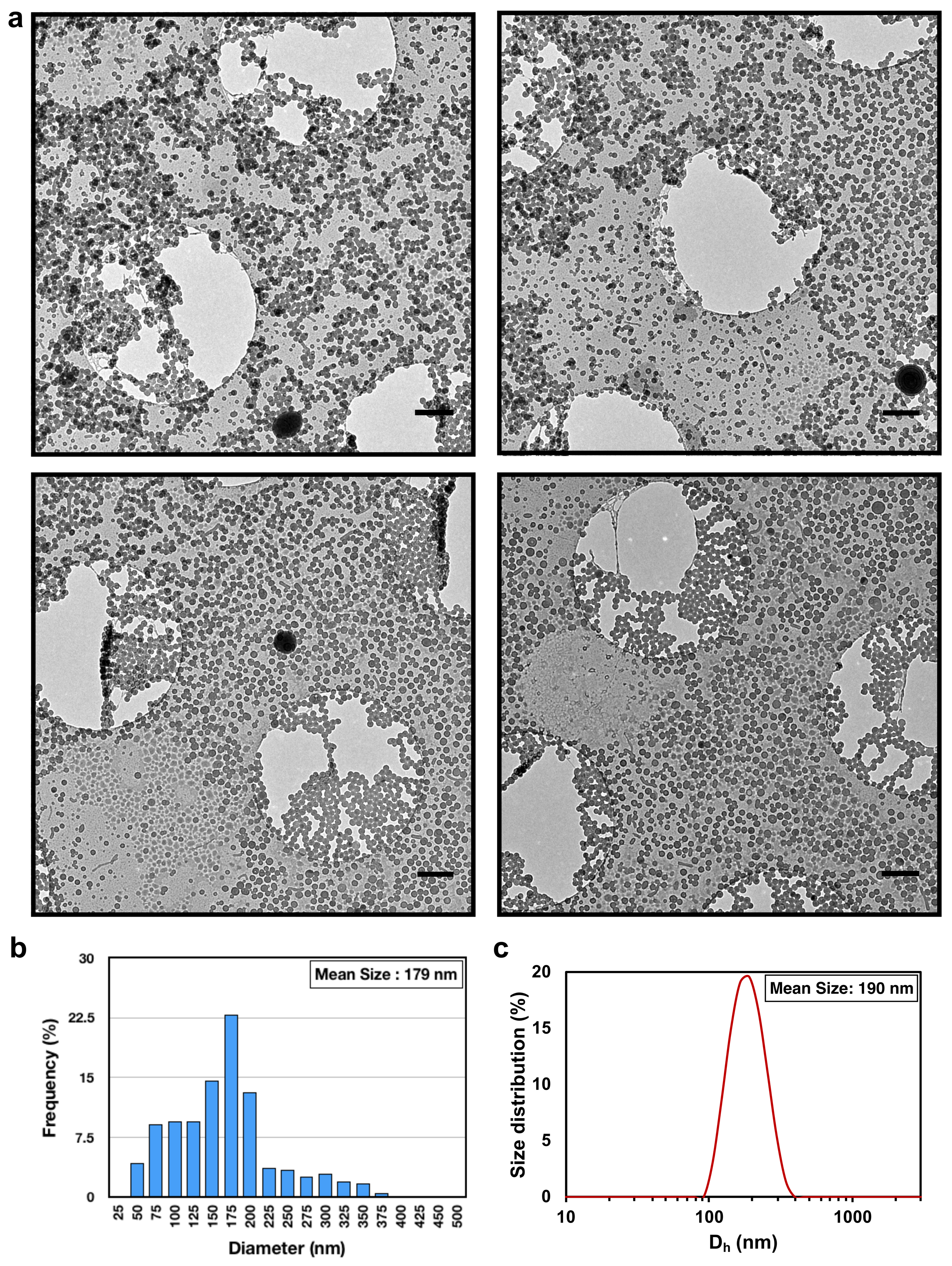
Supplementary Figures and Legends**

**Supplementary Fig. 1 | Size distribution characterisation of lipid vesicles.** (**a**) Cryo-EM characterisation of DOPC and POPC vesicles. Scale bars = 1 µm. (**b**) Size distribution profiles of ~ 500 lipid vesicles from the cryo-EM micrographs analysed using ImageJ software and (**c**) DLS frequency distribution by Intensity.


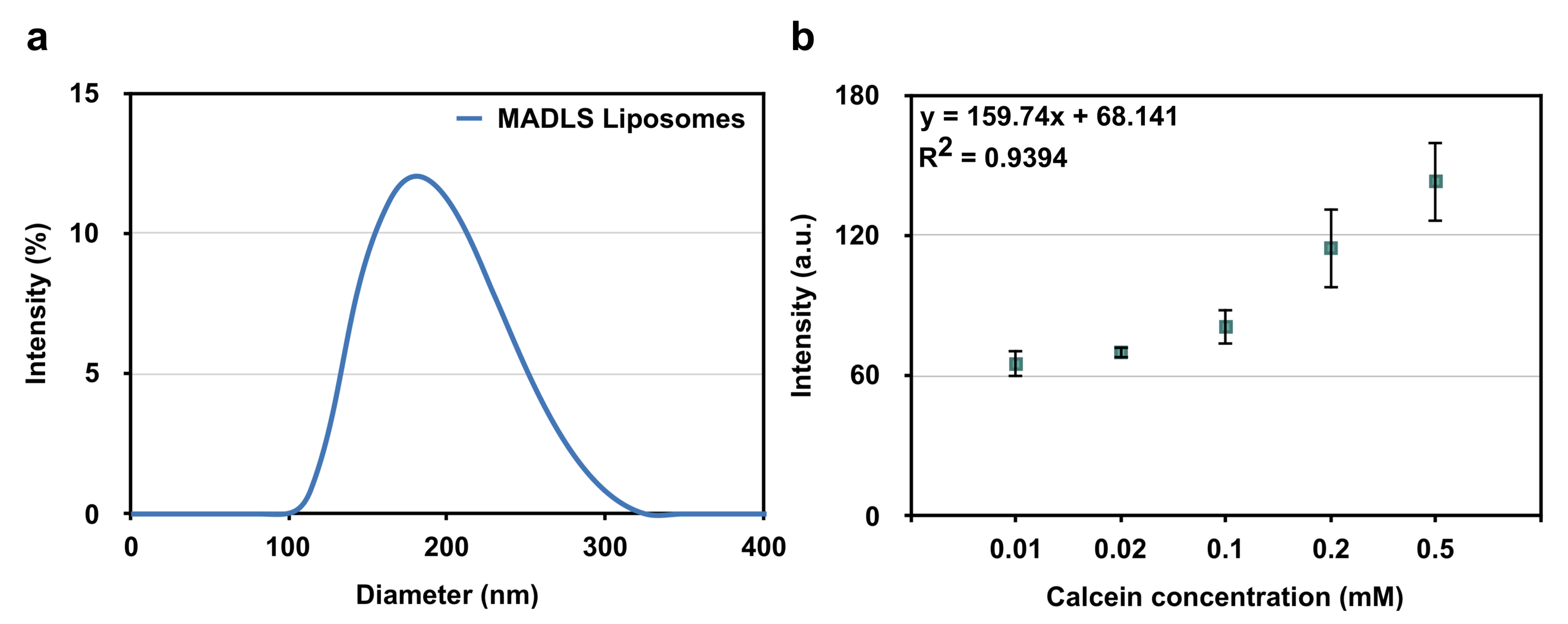


**Supplementary Fig. 2 | Concentration of lipid vesicles in solution.** (**a**) Multi angle dynamic light scattering (MADLS) size distribution of liposomes plotted in a non-logarithmic x-scale and (**b**) calcein calibration curve in fluorescence at 514 nm used to determine to total volume of vesicles in solution. The error bars represent the standard error calculated across the range of samples.

**Supplementary Note 1**

Size distribution is an intrinsic property of colloids, in particular of soft matter assemblies like liposomes. We run a multi angle dynamic light scattering (MADLS) analysis of the vesicle sample. The result confirmed the previous intensity-weighted peak average at around ~ 190 nm (Supplementary Figure 1c) and the measured polydispersity index of the vesicle samples was 0.11 ± 0.015, indicating that the sample is relatively monodisperse. This analysis also confirms and reflects the size distribution obtained with the cryo-EM characterisation (Supplementary Figure 1b). The MADLS plotting is shown in a non-logarithmic x-scale for clarity in Supplementary Figure 2a.


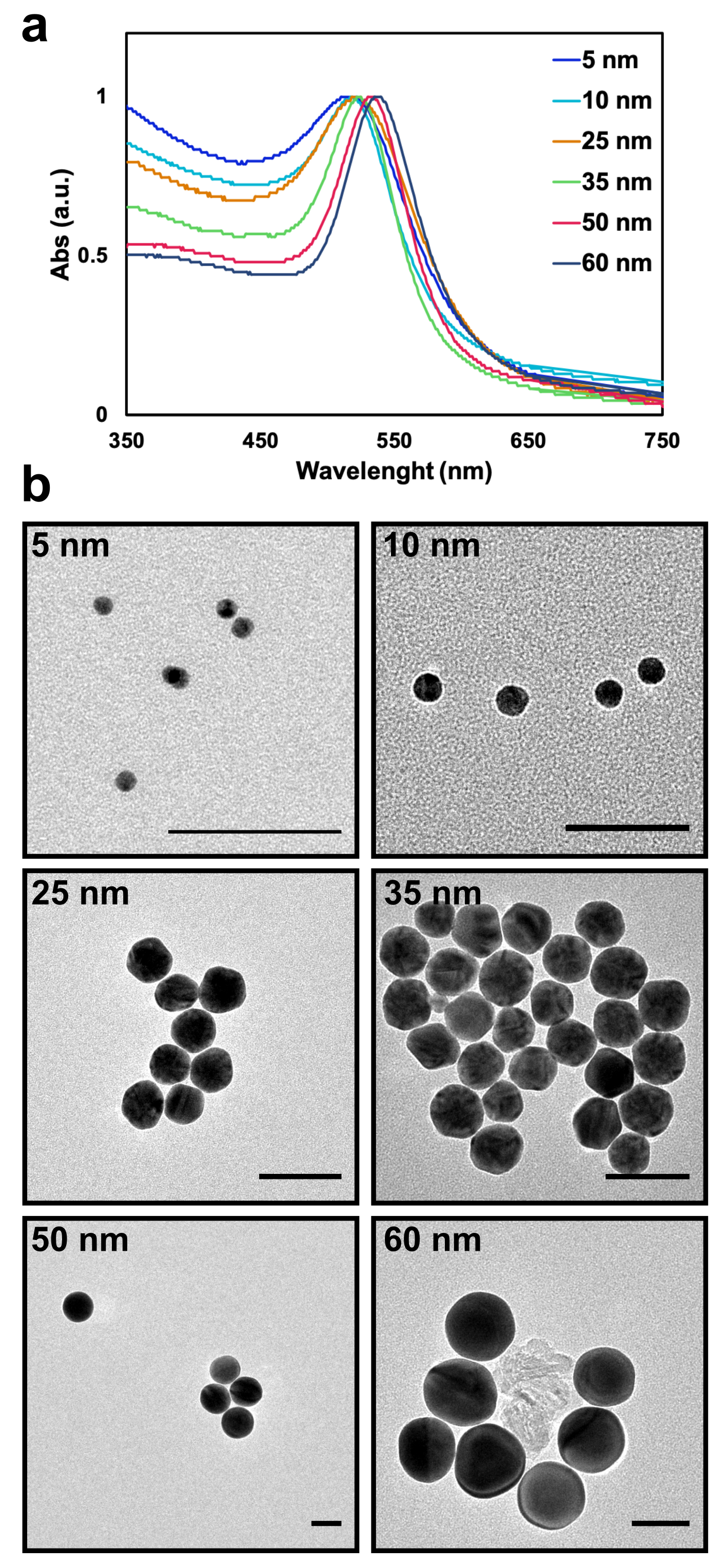


**Supplementary Fig. 3 | AuNPs characterisation.** (**a**) UV-vis absorption­ spectra of the characteristic and progressive plasmon band red shift as the diameter of AuNPs increases with no reduction in UV extinction or unusual peak broadening or shifting, which confirms the absence of AuNPs aggregation. (**b**) TEM micrographs of the employed AuNPs. Scale bar is 50 nm.


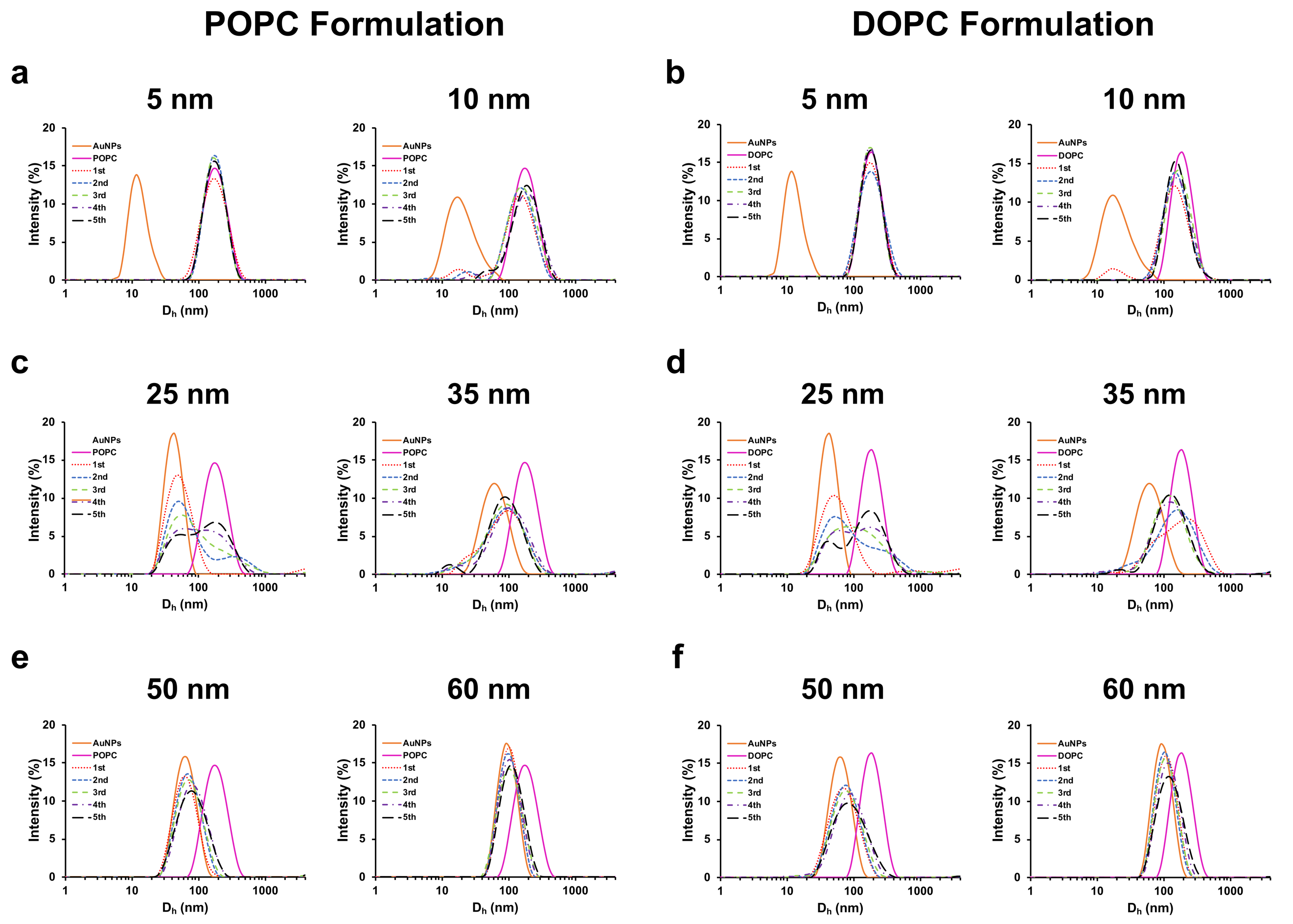


**Supplementary Fig. 4 | Size distribution profiles of the interaction of AuNPs with LUVs.** Size distribution profiles changing from (**a**, **b**) small sizes: 5-10 nm to (**c**, **d**) medium sizes: 25-35 nm and (**e**, **f**) large sizes: 50-60 nm for the POPC and DOPC formulations. All the profiles where obtained by subsequently injecting LUVs within an AuNPs dispersion up to 5 injections (1^st^ injection = dotted red line, 2^nd^ injection= dashed blue line, 3^rd^ injection = dashed green line, 4^th^ injection = dashed violet line and 5^th^ injection = dashed black line). Control samples were obtained by singularly measuring the AuNP (orange) and LUVs (magenta) dispersions.


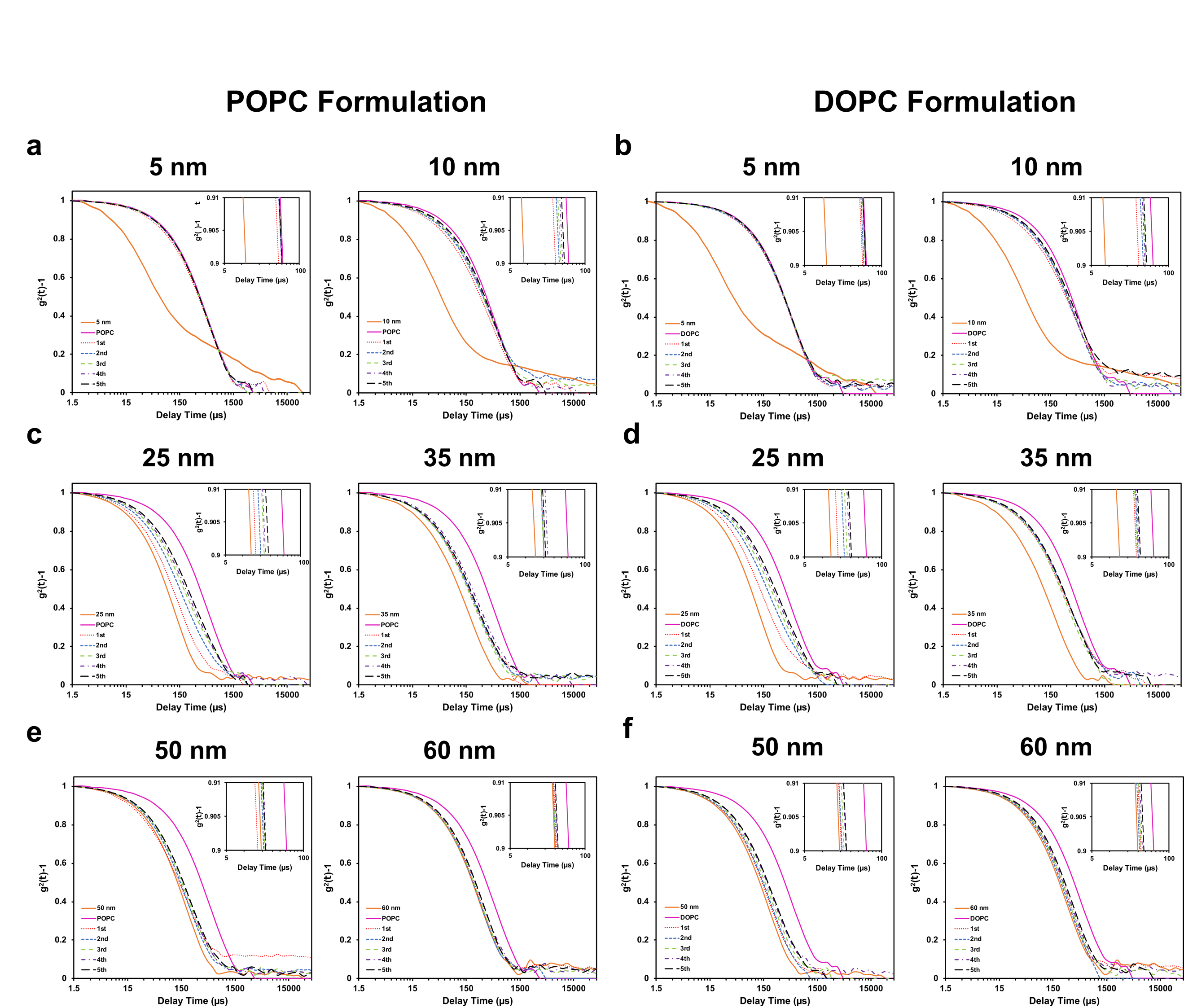
­

**Supplementary Fig. 5 | Autocorrelation function profile of the interaction of AuNPs with LUVs.** Autocorrelation function profiles changing from (**a,b**) small sizes: 5-10 nm to (**c,d**) medium sizes: 25-35 nm and (**e,f**) large sizes: 50-60 nm for the (a,c,e) POPC and (b,d,f) DOPC formulations. All the profiles where obtained by subsequently injecting LUVs within an AuNPs dispersion up to 5 injections (1^st^ injection = dotted red line, 2^nd^ injection= dashed blue line, 3^rd^ injection = dashed green line, 4^th^ injection = dashed violet line and 5^th^ injection = dashed black line). Control samples were obtained by singularly measuring the AuNP (orange) and LUVs (magenta) dispersions.


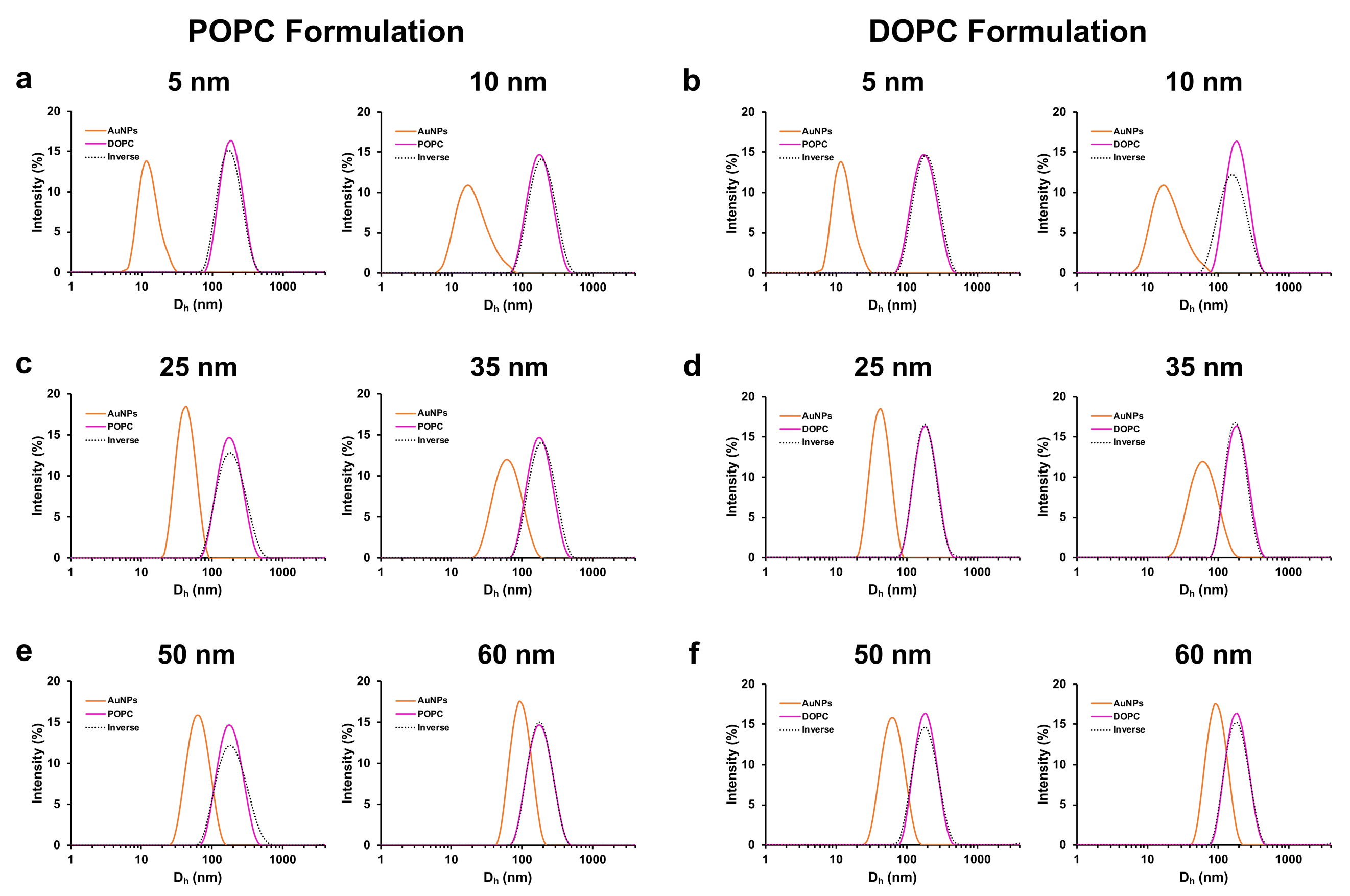


**Supplementary Fig. 6 | Size distribution profiles obtained by injecting AuNP into LUV’s dispersion, inverse injection.** Size distribution profiles of AuNPs (orange), lipid vesicles (magenta) and mixture of AuNPs-lipid vesicles by injecting (**a,b**) small sizes: 5-10 nm, (**c,d**) medium sizes: 25-35 nm and (**e,f**) large sizes: 50-60 nm. Different formulations of liposomes are represented: (a,c,e) POPC and (b,d,f) DOPC.


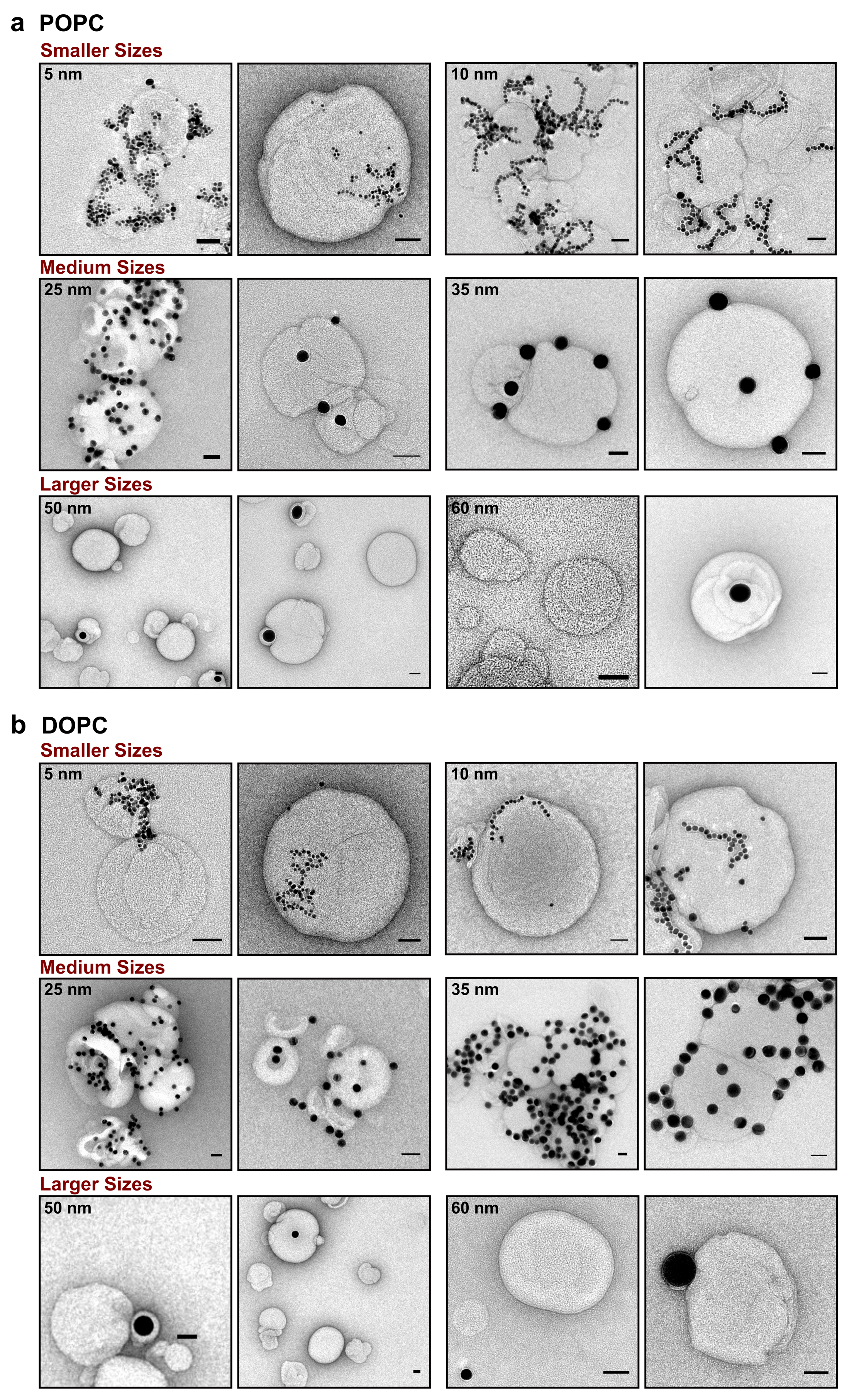


**Supplementary Fig. 7 | TEM micrographs of differently sized** **AuNPs interacting with lipid model membrane.** TEM micrographs of small (5-10 nm), medium (25-35 nm) and large AuNP (50-60 nm) interactions with (a) DOPC and (b) lipid model membrane. Scale bar 50 nm.


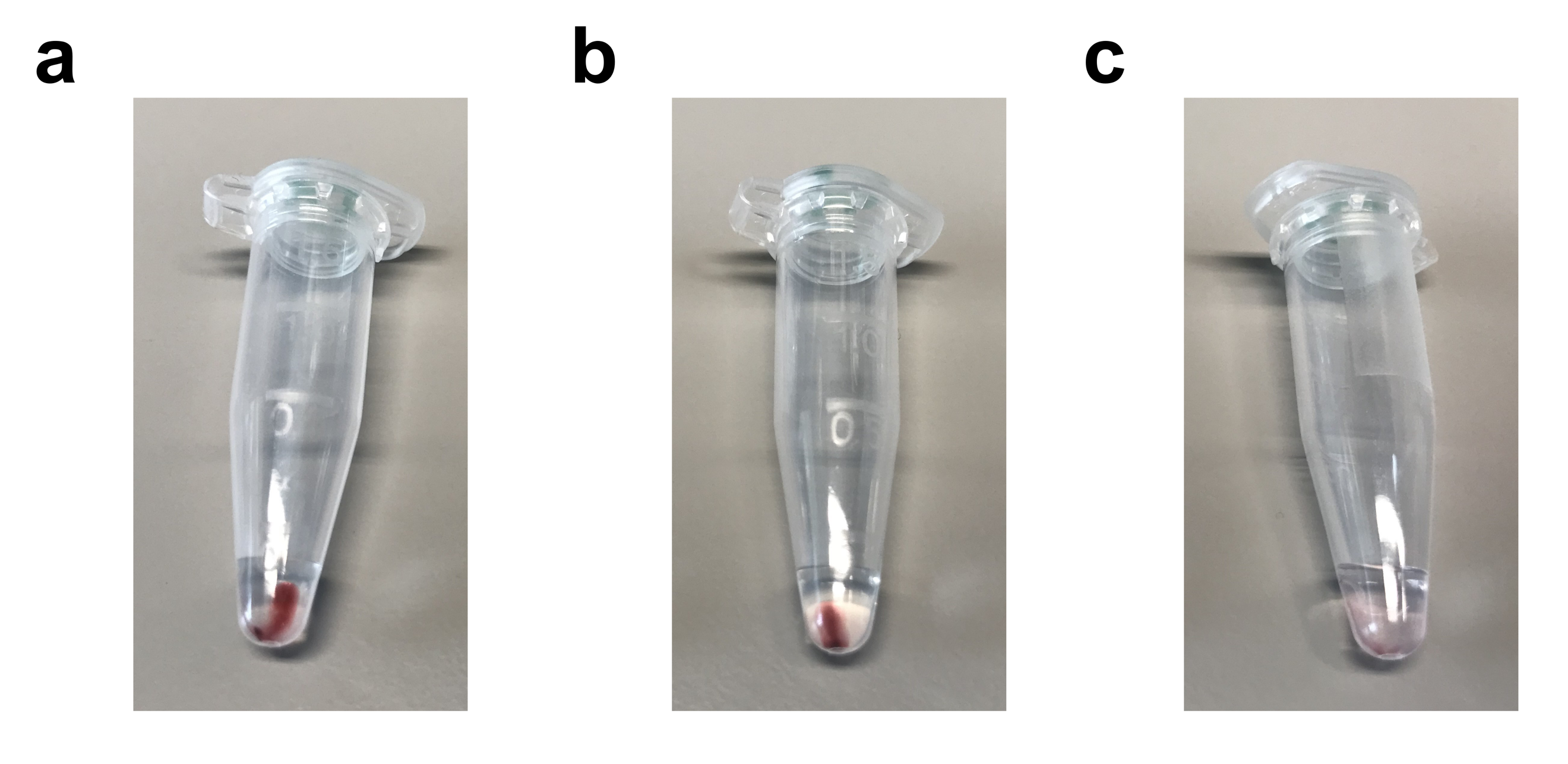


**Supplementary Fig. 8 | AuNPs-LUVs mixture after gentle centrifugation.** The AuNPs-LUVs mixture has been concentrated by gentle centrifugation. Photo of the AuNPs-LUVs sedimentation after centrifugation of the mixture LUV and (**a**) 5 nm, (**b**) 25 nm and (**c**) 60 nm.


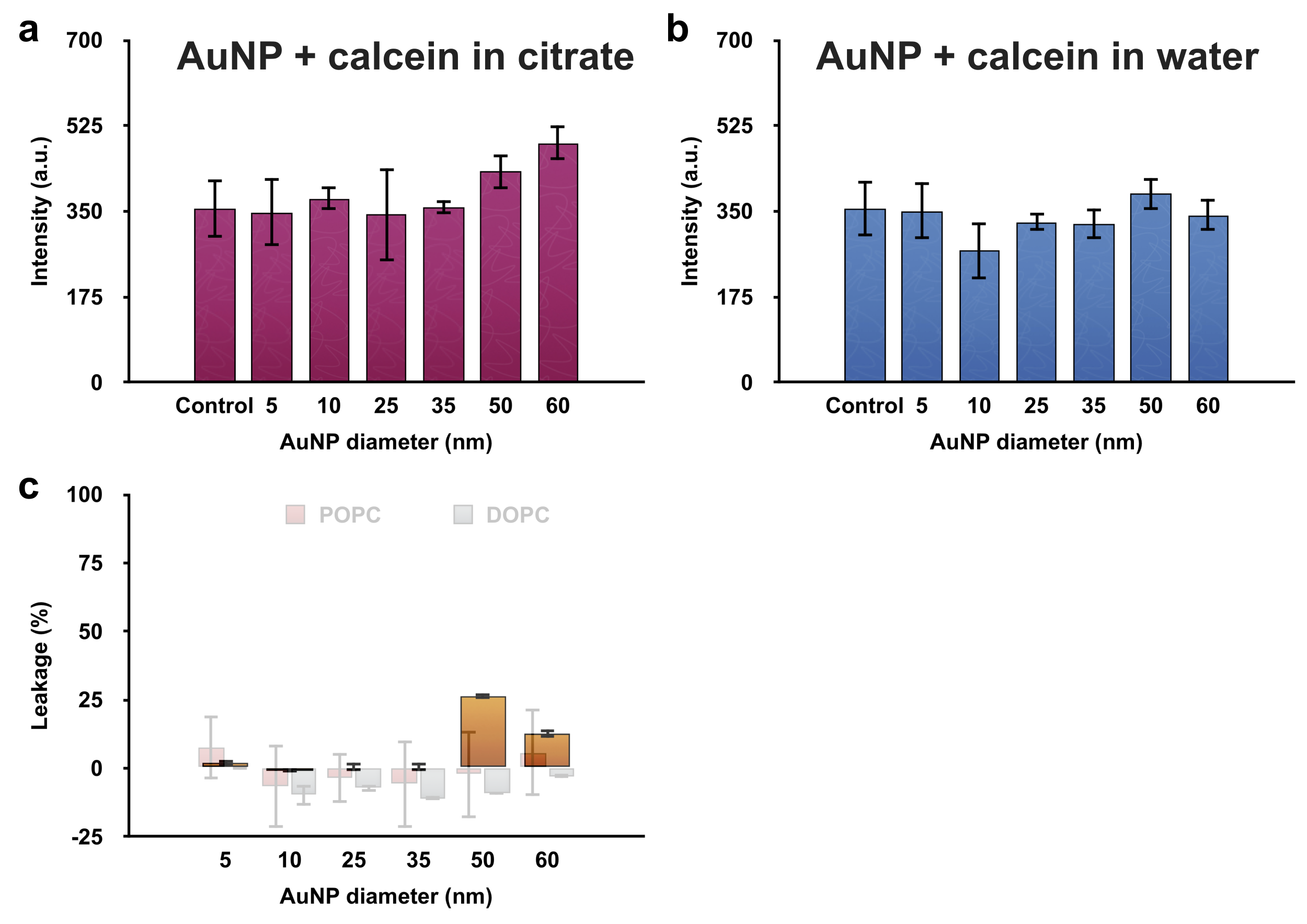


**Supplementary Fig. 9 | Control experiments for the calcein leakage assay.** The potential quenching effect of AuNPs has been investigated by recording the calcein fluorescence in presence of AuNPs only (absence of liposomes). All the AuNPs sizes have been dispersed in the same calcein solution in (**a**) citrate and (**b**) water as dispersant. The control sample refers to calcein solution. (**c**) Leakage assay in water (absence of sucrose). The leakage assay in presence of sucrose is shown as a background. The error bars represent the standard error calculated across the range of samples.


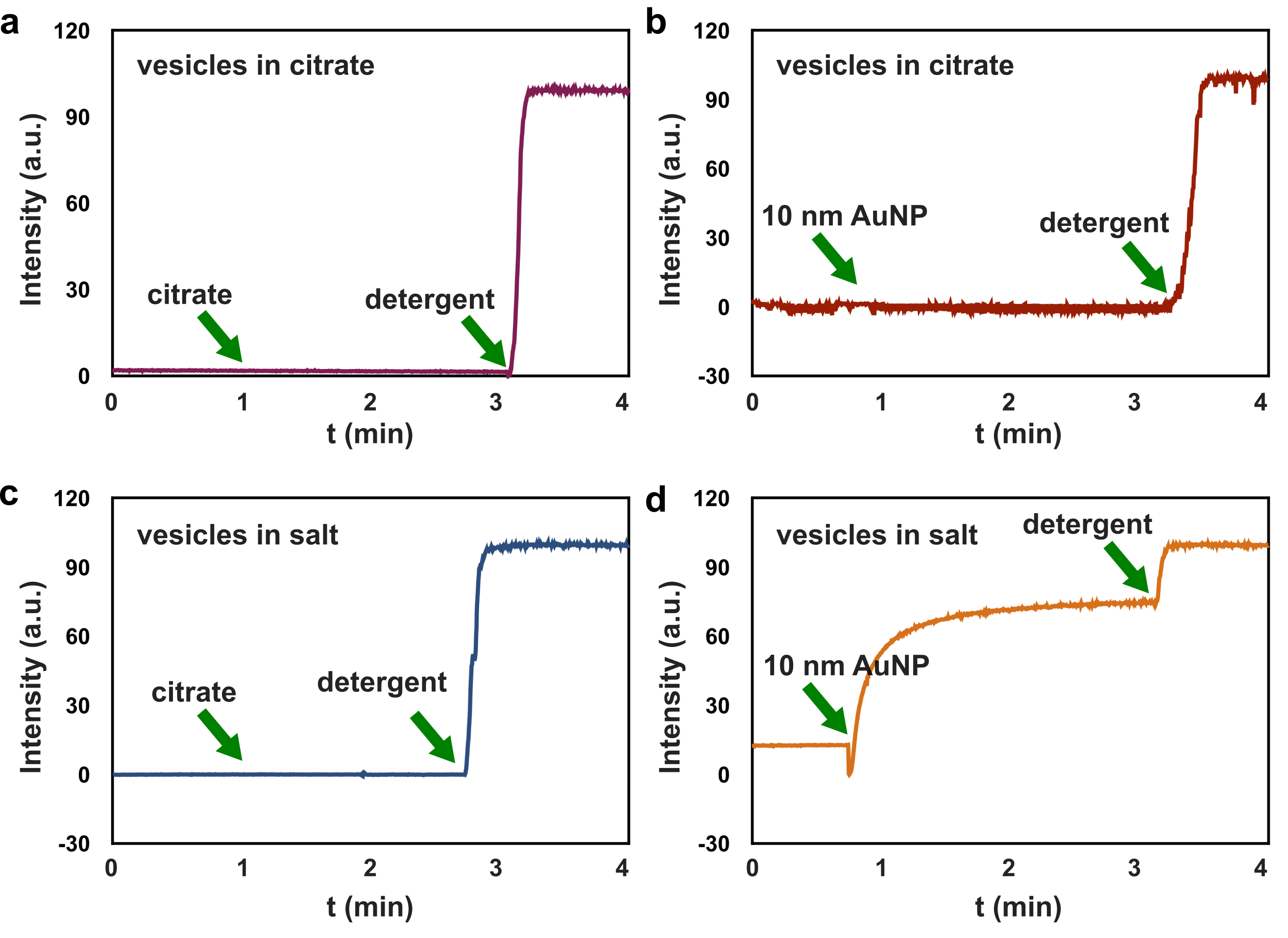


**Supplementary Fig. 10 | Kinetics of calcein release in presence and absence of salt.** Recorded calcein leakage over time from: vesicles dispersed in citrate after the addition of (**a**) citrate and (**b**) 10 nm AuNPs and, vesicles dispersed in citrate after the addition of (**c**) citrate and (**d**) 10 nm AuNPs (positive leakage). The calcein release has been induce in all samples with the addition of triton as detergent.


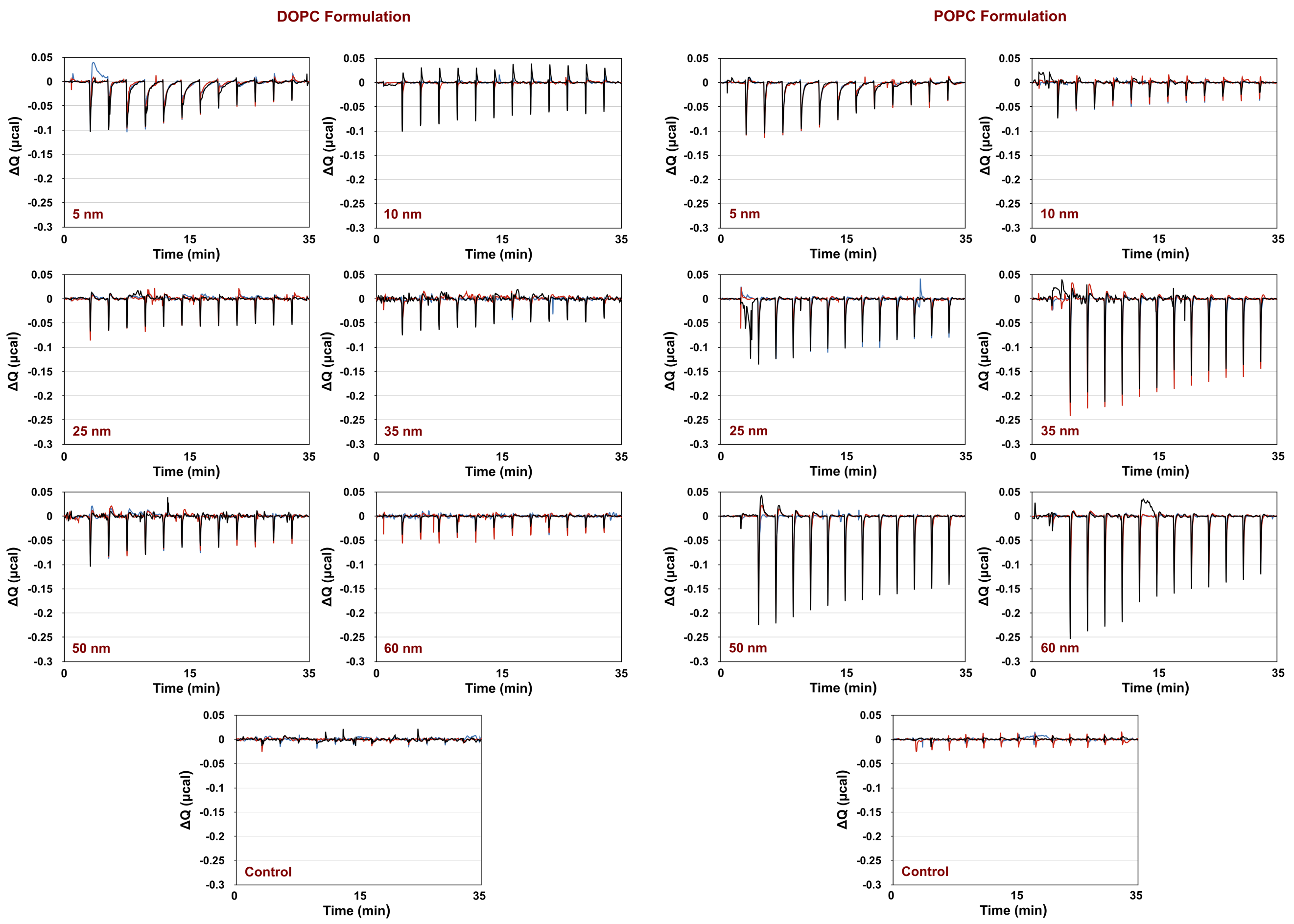


**Supplementary Fig. 11 | ITC heat flows of the AuNPs/liposomes interaction.** The ITC heat flow recordings are show (in µcal per second) for the DOPC (right) and POPC (left) formulation. The data were obtained by titrating the liposomes into the AuNPs dispersions. The control experiment (central bottom) shows the titration of liposome into citrate solution, in absence of AuNPs.
